# Pyroptosis‐Inducing Engineered Microparticles for Cancer Immunotherapy

**DOI:** 10.1002/advs.74886

**Published:** 2026-03-18

**Authors:** Tianli Hao, Zihan Deng, Yufei Liu, Li Liu, Deqiang Deng, Muyang Yang, Yuanyuan Geng, Yao Sun, Beilei Yue, Jonathan F. Lovell, Yushuai Liu, Lisen Lu, Honglin Jin

**Affiliations:** ^1^ College of Biomedicine and Health and College of Life Science and Technology Huazhong Agricultural University Wuhan China; ^2^ NHC Key Laboratory of Tropical Disease Control School of Life Sciences and Medical Technology Hainan Medical University Haikou Hainan China; ^3^ Department of Biomedical Engineering University At Buffalo State University of New York Buffalo New York USA; ^4^ Department of Ophthalmology Union Hospital Tongji Medical College Huazhong University of Science and Technology Wuhan China

**Keywords:** bispecific adapters, cancer immunotherapy, GSDME, microparticles, nanobodies, pyroptosis

## Abstract

Pyroptosis, a form of immunogenic cell death, can remodel the immunosuppressive tumor microenvironment; however, its clinical translation is challenged by difficulties in targeted induction and biosafety concerns. In this study we discovered that geldanamycin (GA) can interacts specifically with arginine 207 (R207) in caspase‑3, and efficiently cleaved caspase‐3, significantly inducing high‐efficiency pyroptosis. Although high‐dose GA promoted pyroptosis and antitumor immunity, physiological toxicity and the induction of resistance mediated by immune checkpoints including PD‐L1 and CD47 limited therapeutic efficacy. To address this, we developed biomimetic dual‐targeting microparticles(MPs)functionalized with anti‐PD‐L1 and anti‐SIRPα nanobodies. This strategy synergized targeted pyroptosis induction with dual immune checkpoint blockade, achieving complete tumor regression, reduced physiological toxicity, and induced a potent effector immune response in murine cancer models, presenting a promising combinatorial approach for lung cancer immunotherapy.

## Graphical abstract

Engineered microparticles co‐delivering geldanamycin and dual nanobodies induce targeted pyroptosis and block PD‐L1 and CD47 pathways, reprogramming the tumor microenvironment and achieving potent antitumor immunity in lung cancer models with minimal toxicity.

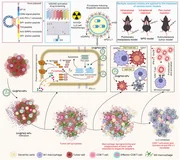

## 1 Introduction

Cancer immunotherapy, particularly immune checkpoint blockade (ICB), has revolutionized oncology by harnessing the host immune system to eliminate tumors [1, 2]. However, the efficacy of ICB is often limited in immunologically “cold” tumors [3]. These “cold” tumors are characterized by tumor immunosuppressive microenvironment (TIME), a lack of sufficient immune cell infiltration such as T cells, and a low tumor mutational burden, making them difficult for the immune system to recognize [3, 4]. In contrast, “hot” tumors exhibit high immunogenicity and substantial immune cell infiltration, particularly with an abundance of cytotoxic T cells, rendering them generally more responsive to immunotherapy [4, 5]. Inducing immunogenic cell death (ICD) represents a promising strategy to convert these “cold” tumors into “hot” ones, thereby enhancing their response to immunotherapy [6, 7]. Pyroptosis, a highly inflammatory and immunogenic form of programmed cell death mediated by gasdermin (GSDM) family proteins, has thus attracted considerable interest [8, 9, 10]. This process is executed when gasdermin proteins form pores in the plasma membrane, leading to cell lysis and the release of pro‐inflammatory cytokines and damage‐associated molecular patterns (DAMPs) [11]. These released signals reshape the TIME by promoting the recruitment and activation of immune cells, particularly through driving dendritic cell (DC) maturation and cytotoxic T lymphocyte (CTL) infiltration, which ultimately reverses the immunosuppressive state [12, 13]. Notably, inducing pyroptosis in a relatively small fraction of tumor cells can eradicate entire tumors, underscoring its potential as a powerful amplifier of anti‐tumor immunity [14, 15].

Despite this promise, the clinical translation of pyroptosis‐based therapies faces significant hurdles. Current pyroptosis‐inducing agents, such as conventional chemotherapeutics, often lack specificity and induce severe off‐target toxicity [16]. Furthermore, even when pyroptosis is successfully induced, it may still compensatorily recruit and activate immunosuppressive cells within the tumor immune microenvironment (TIME), thereby eliciting adaptive immune resistance [13, 17]. This feedback mechanism may dampen immune activation and facilitate tumor immune escape. Therefore, there is a need for strategies that not only induce robust pyroptosis but also concurrently counteract these immune evasion pathways.

To address the delivery challenges associated with pyroptosis inducers, biomimetic nanocarriers have emerged as promising vehicles [18, 19]. Among these, microparticles (MPs) derived from irradiated tumor cells are particularly attractive. Radiation‐induced MPs inherently mimic key aspects of ICD and possess a natural ability to reprogram macrophages, coupled with high drug‐loading efficiency and suitability for genetic engineering [20, 21]. As such, they serve as an excellent platform for co‐delivering therapeutic agents directly to the tumor site. However, while serving as effective carriers, these MPs alone typically exhibit limited direct anti‐tumor efficacy. Their primary advantage lies in their ability to be engineered as multi‐functional systems that can synergize targeted drug delivery with immunomodulation [22, 23]. With a potent payload and complementary mechanisms to overcome adaptive immune resistance, their therapeutic efficacy can be fully improved.

**SCHEME 1:**
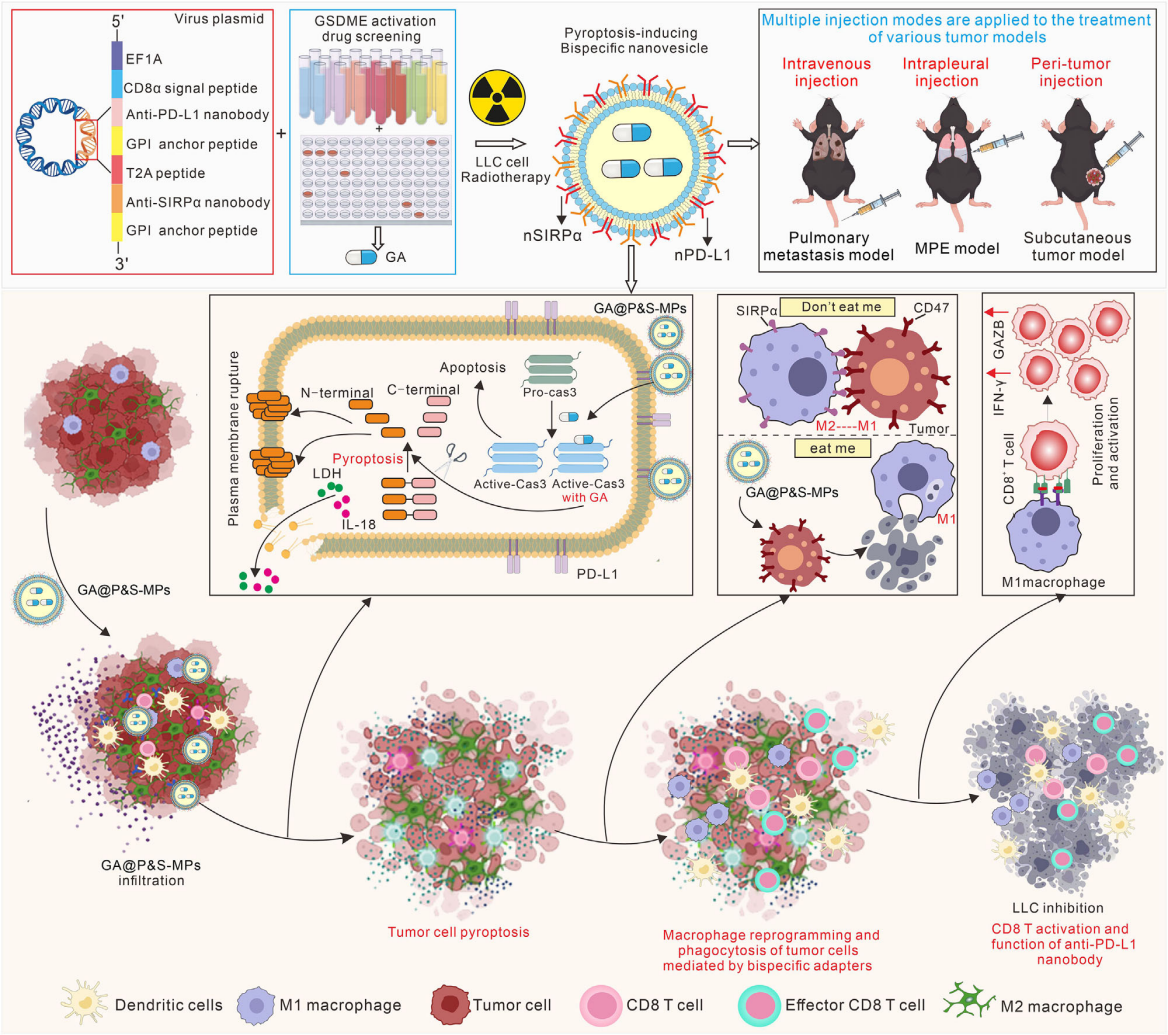
Application and Anti‐Tumor Mechanisms of GA@P&S‐MPs in Lung Cancer Treatment. This schematic illustrates the mechanism of action and therapeutic application of GA@P&S‐MPs in lung cancer. The geldanamycin (GA)‐loaded nanoparticles, functionalized with bispecific adapters such as anti‐PD‐L1 and anti‐SIRPα nanobodies, enable dual targeting of both tumor cells and macrophages. GA, promotes the cleavage of caspase‐3 and facilitates molecular glue‐like interactions between cleaved caspase‐3 and GSDME, triggering GSDME cleavage and pyroptosis. Furthermore, GA reprograms M2‐type macrophages. By bridging tumor cells and macrophages, GA@P&S‐MPs enhance phagocytosis and antigen presentation by innate immune cells. They also block critical immune checkpoint pathways including PD‐L1/PD‐1 and CD47/SIRPα, thereby activating both innate and adaptive immunity and ultimately achieving potent antitumor responses across diverse lung cancer models.

Here, we identified geldanamycin (GA) as a potent and specific inducer of GSDME‐dependent pyroptosis. We demonstrate that GA functions uniquely by directly binding to caspase‐3, leading to efficient pyroptotic cell death. In vivo, GA monotherapy not only inhibited tumor growth and metastasis but also favorably remodeled the TIME by promoting M1 macrophage polarization, DC maturation, and CD8^+^ T cell infiltration. However, consistent with the challenges outlined above, GA treatment also induced the adaptive upregulation of PD‐L1 and CD47, highlighting the necessity for a combination approach. To overcome this limitation, we engineered a biomimetic, dual‐targeting microparticle system (GA@P&S‐MPs). This system was constructed using MPs derived from irradiated tumor cells engineered to express surface‐anchored anti‐PD‐L1 and anti‐SIRPα nanobodies, which were subsequently loaded with GA. This design aims to synergize targeted pyroptosis induction via GA with simultaneous blockade of the PD‐1/PD‐L1 and CD47/SIRPα immune inhibitory axes. Our results demonstrate that GA@P&S‐MPs achieve superior anti‐tumor efficacy, profound immune microenvironment remodeling, and abscopal effects while mitigating the systemic toxicity associated with high‐dose GA (Scheme 1). This work presents a novel and potentially translatable combinatorial strategy for effective lung cancer immunotherapy by concurrently activating ICD and dismantling key mechanisms of immune suppression.

## 2 Results

### 2.1 Identification of GSDME as a Key Regulator With Prognostic and Immunomodulatory Significance in Lung Cancer

To systematically evaluate the role of GSDM family members in lung cancer pathogenesis and their potential as therapeutic targets, we first interrogated the TCGA database to compare the expression profiles of GSDMA, GSDMB, GSDMC, GSDMD, and GSDME between lung tumor tissues and adjacent normal samples. Our analysis revealed that GSDME exhibited the most pronounced differential expression among all GSDM genes, with significantly elevated levels in tumor compared to normal tissue (Figure S1A,C, Supporting information). Although Kaplan‐Meier survival analysis indicated that the expression of GSDME itself was not directly correlated with patient prognosis (Figure S1B, Supporting information), its marked overexpression in tumors suggested a potential functional role in lung cancer biology. Given that GSDME‐mediated pyroptosis requires proteolytic activation, we further investigated the prognostic relevance of upstream regulators and activators of GSDME. Intriguingly, several genes involved in the cleavage and regulation of GSDME activity—including caspase‐4 [24], caspase‐5, ZDHHC5 [25], and other modifiers—showed significant associations with overall survival in patients with lung cancer (Figure S1D–H, Supporting information). These findings imply that while GSDME expression alone may not predict survival outcomes, the functional state of GSDME—modulated by its regulatory molecules—is critically linked to patient survival.

To explore whether GSDME activity influences tumor‐immune crosstalk, we performed single‐cell RNA sequencing analysis using the GEO dataset (GSE148071). Unsupervised clustering identified major cell types within the lung tumor microenvironment (Figure S1I, Supporting information), with canonical marker genes confirming cell identity (Figure S1J, Supporting information). Subclustering of malignant cells further revealed heterogeneous expression of GSDME, allowing stratification into GSDME^high^ populations (Figure S1K, Supporting information). Within these, we distinguished two distinct subsets (GSDME^high^CASP4^high^ and GSDME^high^CASP4^low^ clusters) based on caspase‐4 (CASP4) expression. Subsequent ligand‐receptor interaction analysis demonstrated that these two subpopulations exhibited markedly different communication patterns with immune cells (Figure S1L,M, Supporting information). Specifically, the GSDME^high^CASP4^high^ cluster showed enhanced interactions with various immune subsets through distinct ligand‐receptor pairs, suggesting a more immunomodulatory phenotype. These results highlight that not only is GSDME expression elevated in lung cancer, but also shapes intercellular communication within the tumor microenvironment. In summary, our integrated bioinformatic and single‐cell analyses establish GSDME as a central player in lung cancer, with its activation state—rather than mere expression—being closely tied to patient prognosis and immune regulation. These insights provide a compelling rationale for targeting GSDME activation pathways as a novel therapeutic strategy in lung cancer.

### 2.2 Identification of Small‐Molecule Compounds Capable of Inducing Pyroptosis

**FIGURE 1:**
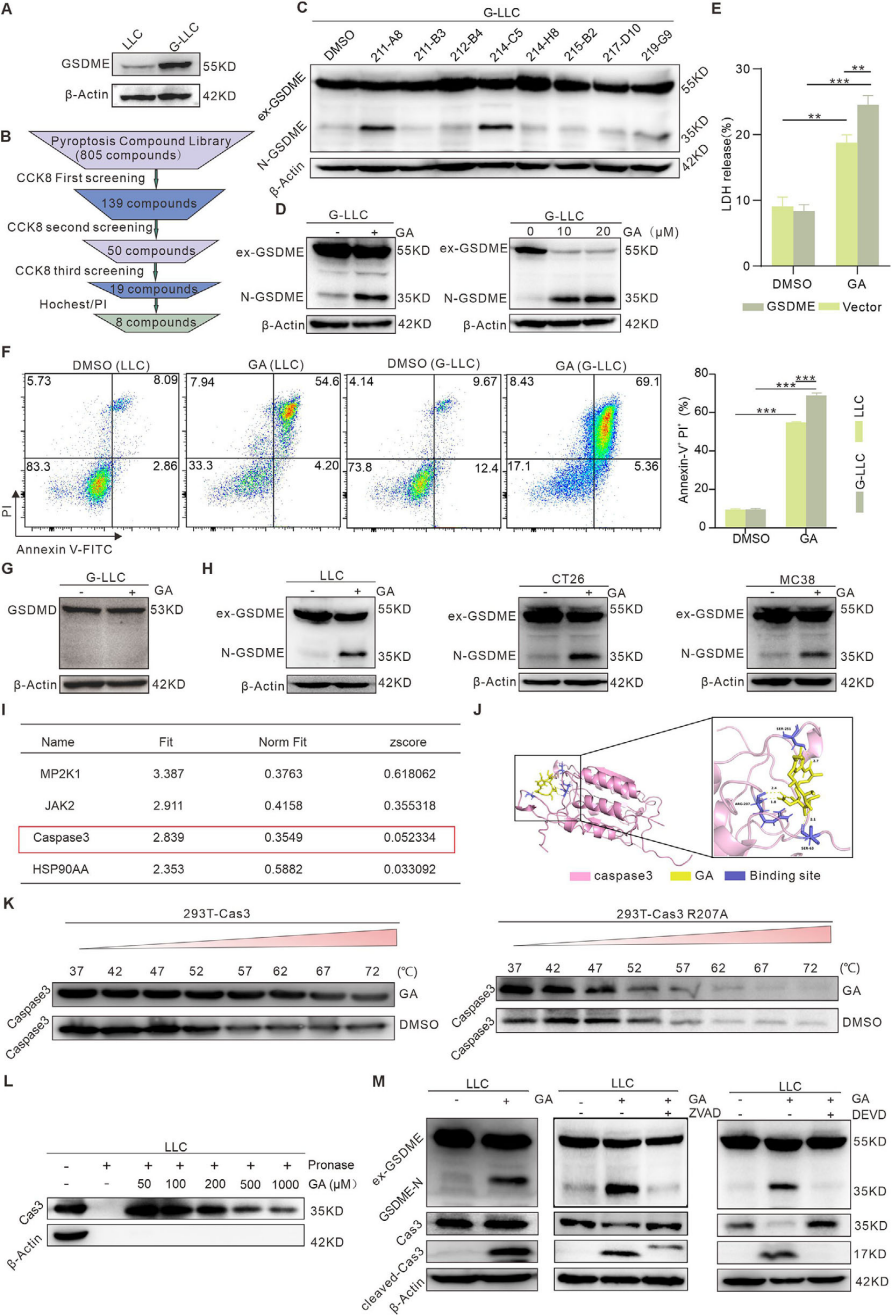
Identification of small‐molecule drugs that can induce efficient pyroptosis and the mechanisms by which they induce pyroptosis in cells. (A) Western blot analysis of GSDME expression in LLC and G‐LLC (Transfecting plasmids with high expression of GSDME) tumor cells. (B) Strategies for screening small‐molecule drugs that efficiently induce pyroptosis. (C) Western blot of N‐GSDME (N terminal of GSDME) and ex‐GSDME (Full‐length GSDME) expression in G‐LLC after indicated treatment. (D) Validation of the efficiency of GA in cleaving GSDME in G‐LLC cells via western blotting and validate the efficiency of GA in cleaving GSDME in G‐LLC tumor cells at different concentrations. (E) Elisa assay was used to detect the LDH level from the supernatant. n = 3 biologically independent samples. (F) Use flow cytometry combined with Annexin‐V antibody and PI dye to assess the effects of GA on apoptosis in LLC and G‐LLC cells after 6 h of incubation. n = 3 biologically independent samples. (G) Validation of the effect of GA on the cleavage of GSDMD in G‐LLC cells using western blotting. (H) Validation of the effect of GA on the cleavage of GSDME in various tumor cells with high GSDME expression. (I) The potential target candidates for GA predicted by PharmaMappery Server. (J) A representative image of analysis of the binding site of GA with caspase‐3 by using PyMOL. (K) Analysis of the thermal stability of wild‐type and mutant caspase‐3 in the presence or absence of GA by using Cell thermal shift assay (CETSA). (L) Analysis of the proteolytic degradation stability of wild‐type caspase‐3 in the presence or absence of GA by using Drug affinity reactive target stability assay (DARTs). (M) Analysis of the role of cleaved Caspase‐3 in cleaving GSDME in LLC cells by using western blotting. Data are presented as mean ± SEM, and the statistical significance was analyzed via one‐way ANOVA. *p < 0.05, **p < 0.01, ***p < 0.001, and NS: not significant.

To identify compounds that trigger pyroptosis in tumor cells expressing high levels of GSDME, we first established a stably transfected mouse Lewis lung carcinoma (LLC) cell line with elevated GSDME expression (designated G‑LLC; Figure 1A). Using a focused library of pyroptosis‐related compounds, we performed three rounds of CCK‑8 viability screening and identified 19 candidates that significantly reduced viability in G‑LLC cells (Figure 1B and Figure S2, Supporting Information). Subsequent confocal imaging combined with propidium iodide (PI) staining after 6 h of drug exposure was employed to detect rapid plasma membrane rupture, a hallmark of pyroptosis. This narrowed the list to 8 compounds that induced strong PI uptake (Figure S3A, Supporting Information). To assess whether these candidates specifically cleaved GSDME to drive pyroptosis, we performed Western blot analysis. This identified geldanamycin (GA, compound 214‑C5) as the most consistent and potent inducer of GSDME cleavage (Figure 1C). Dose–response experiments confirmed that GA induced GSDME fragmentation in a concentration‐dependent manner (Figure 1D). Consistent with these results, GA treatment also led to significant lactate dehydrogenase (LDH) release (Figure 1E), indicative of lytic cell death. Flow cytometry analysis further revealed that GA induced substantial cell death in both G‑LLC and parental LLC cells, with markedly enhanced efficacy in GSDME‐high backgrounds, and was associated with classical apoptotic and pyroptotic markers (Figure 1F). Live‐cell imaging of LLC cells treated with GA further demonstrates that GA induces cell death through pyroptosis (Video S1, Supporting Information). Notably, GA did not induce cleavage of GSDMD (Figure 1G), confirming its specificity toward the GSDME pathway. We extended these observations to several human and murine tumor cell lines endogenously expressing high levels of GSDME (Figure S3B,C, Supporting Information). Across these models, GA consistently triggered robust GSDME cleavage (Figure 1H), underscoring its broad applicability as a pyroptosis‐inducing agent.

We next investigated the functional role of GSDME in GA‐induced pyroptosis. Efficient knockdown of GSDME in LLC cells was confirmed by Western blot (shG‑LLC; Figure S3D, Supporting Information). Compared with normal LLC cells and GSDME‑overexpressing G‑LLC cells, shG‑LLC cells exhibited significantly reduced LDH release upon GA treatment (Figure S3E, Supporting Information). Correspondingly, Hoechst/PI double‐staining assay and flow cytometry analysis showed that GA‐induced cell death was substantially attenuated in shG‑LLC cells, whereas it was enhanced in G‑LLC cells (Figure S3F,G, Supporting Information). These results demonstrate that GSDME expression is necessary for GA to elicit full pyroptotic death. To elucidate the mechanism underlying GA‐induced GSDME cleavage, we used the PharmMapper server [34, 35, 36] for reverse pharmacophore mapping and identified caspase‑3 as a top predicted binding target of GA—consistent with its known role in GSDME activation (Figure 1I). HSP90AA1, a previously reported GA target, was also identified, supporting the reliability of the screening [26]. Western blot analysis further confirmed that GA's ability to cleave caspase‑3 is independent of HSP90 activity regulation (Figure S3H, Supporting Information). Molecular docking and structural simulation using PyMOL revealed that GA interacts specifically with arginine 207 (R207) in caspase‑3 (Figure 1J), suggesting direct binding. Molecular dynamics simulations revealed that the GA‐bound wild‐type Caspase‑3 exhibited greater structural stability than the R207A mutant, as assessed by root mean square deviation (RMSD) (Figure S3I, Supporting Information). To validate this interaction, we performed cellular thermal shift assays (CETSA) and drug affinity responsive target stability (DARTS) assays. GA treatment markedly stabilized caspase‑3 against thermal denaturation in wild‐type 293T cells, in contrast to cells expressing the caspase‑3 R207A mutant (Figure 1K and Figure S3J, Supporting Information), indicating that R207 is essential for GA binding. Similarly, GA protected caspase‑3 from proteolytic degradation by pronase in the DARTS assay (Figure 1L), further confirming direct binding. Functionally, GA‐induced caspase‑3 activation correlated closely with GSDME cleavage (Figure 1M). Pretreatment with the pan‐caspase inhibitor Z‑VAD or the caspase‑3‑specific inhibitor DEVD completely abolished GA‐mediated GSDME cleavage and pyroptosis. Collectively, these results demonstrate that GA binds directly to caspase‑3 at R207, stabilizes the protein, and thereby promotes GSDME cleavage in an HSP90‑independent manner. This mechanism leads to potent and specific pyroptosis in tumor cells expressing GSDME.

### 2.3 Geldanamycin Inhibits Tumor Growth and Metastasis by Remodeling the Immune Microenvironment In Vivo

**FIGURE 2:**
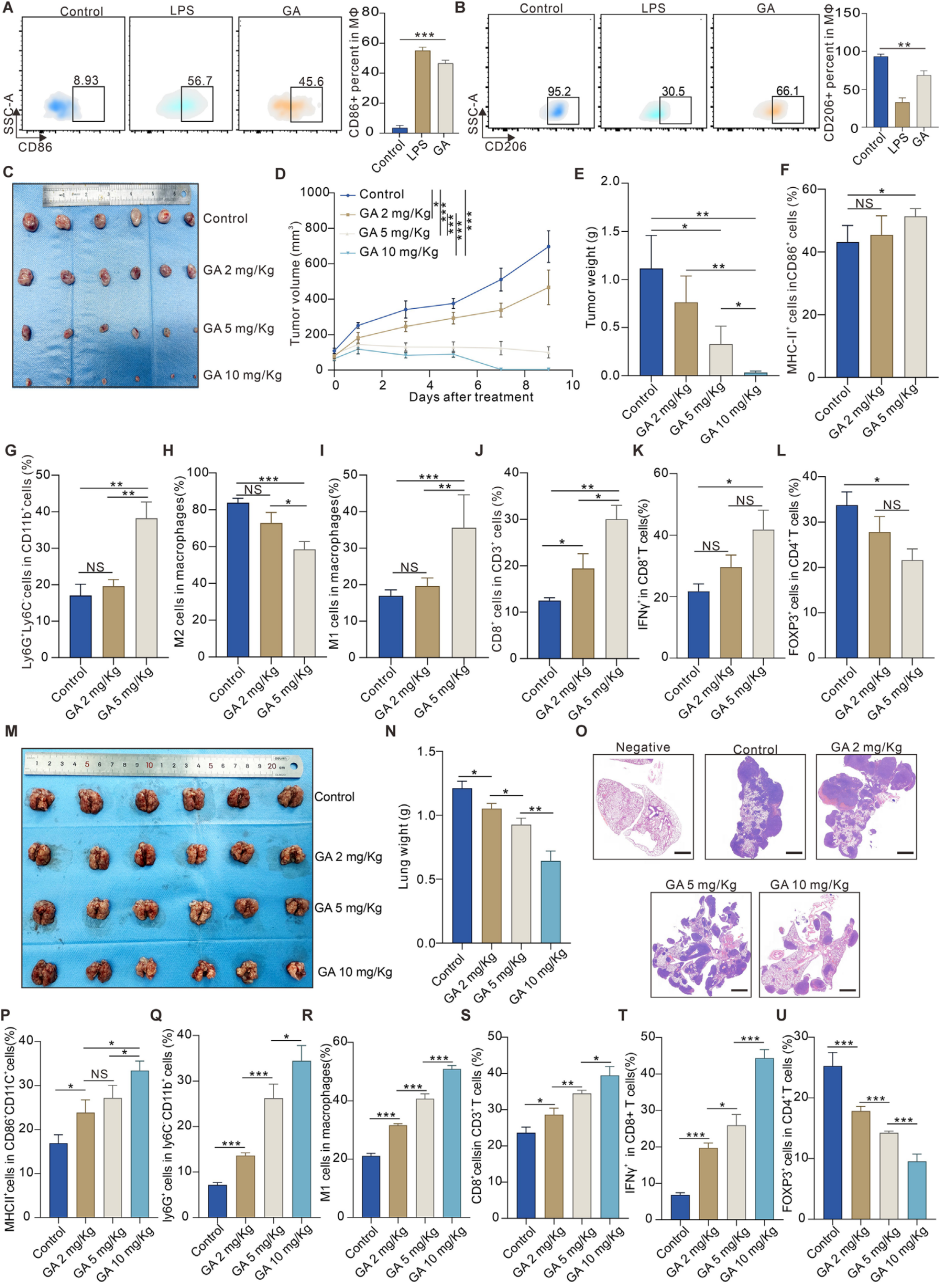
Validation of the therapeutic mechanisms of GA in treating LLC subcutaneous tumors or lung metastases in vivo. (A,B) Validation of the ability of GA to induce the formation of M1 macrophages (A) and inhibit the formation of M2 macrophages in BMDMs by using flow cytometry (B). n = 3 biologically independent samples. (C) Represent bright‐field imaging to assess the effects of intratumoral injection of different concentrations of GA on the growth of LLC subcutaneous tumor models. n = 6 mice per group. (D) Analysis of the effects of intratumoral injection of different concentrations of GA on the growth of LLC subcutaneous tumor models by measuring tumor sizes every other days. n = 6 mice per group. (E) Characterization of the effects of indicated concentration of GA on the weight of LLC subcutaneous tumors in mice. n = 6 mice per group. (F–L) Effects of the indicated concentration of GA therapy on the proportion and number of immune cell subsets in TIME when reaching the endpoint of the tumor prevention experiment. n = 5 mice per group. (F) Statistical analysis of the proportion of MHC‐II^+^CD86^+^DCs in total tumor‐infiltrating DCs. (G) Statistical analysis of the proportion of Ly6G^+^Ly6c^−^ neutrophils within the total population of CD11b^+^ tumor‐infiltrating myeloid cells. (H) Statistical analysis of the proportion of M2 macrophages in the total population of tumor‐infiltrating macrophages. (I) Statistical analysis of the proportion of M1 macrophage in the total tumor‐infiltrating macrophage population. (J) Statistical analysis of the proportion of CD8^+^ T cells in the total population of tumor‐infiltrating CD3^+^ T cells. (K) Statistical analysis of the proportion of IFN‐γ^+^ T cells in the total population of tumor‐infiltrating CD8^+^ T cells. (L) Statistical analysis of the proportion of FoxP3^+^ T cells in the total population of tumor‐infiltrating CD8^+^ T cells. (M) Physical images of in vitro lung metastasis in LLC bearing mice treated with the indicated concentration of GA. n = 6 mice per group. (N) Characterization of the effects of indicated concentration of GA on the weight of LLC lung metastasis model in mice. (O) Assessment of the pulmonary metastasis of LLC cells in vivo after treatment with indicated concentrations of GA by using hematoxylin and eosin staining. The scale bar is 1 mm. (P–U) Effects of the indicated concentration of GA therapy on the proportion and number of immune cell subsets in lung TIME when reaching the endpoint of the tumor prevention experiment. n = 5 mice per group. (P) Statistical analysis of the proportion of MHC‐II^+^CD86^+^DCs in lung TIME. (Q) Statistical analysis of the proportion of Ly6G^+^Ly6c^−^ neutrophils in the total CD11b^+^ myeloid cell of lung TIME. (R) Statistical analysis of the proportion of M1 macrophage in lung TIME. (S) Statistical analysis of the proportion of CD8^+^ T cells in CD3^+^ T cells of lung TIME. (T) Statistical analysis of the proportion of IFN‐γ^+^ T cells in the total population of CD8^+^ T cells of lung TIME. (U) Statistical analysis of the proportion of FoxP3^+^ T cells in the total CD8^+^ T cell population in the lung TIME. Data are presented as mean ± SEM, and statistical significance was analyzed via one‐way ANOVA. *p < 0.05, **p < 0.01, ***p < 0.001, and NS: not significant.

Having established the potent capacity of GA to induce pyroptosis via caspase‐3–GSDME signaling in vitro, we next evaluated the antitumor efficacy of GA and immunomodulatory functions in vivo. We first assessed the direct effect of GA (A concentration of 10 µM of GA has been reported to be non‐toxic to macrophages, which was used for subsequent experiments [27]) on macrophage polarization using bone marrow‐derived macrophages (BMDMs). Flow cytometric analysis revealed that GA treatment significantly promoted the polarization of M1 macrophages (Figure 2A) while concurrently suppressing the formation of M2 macrophages (Figure 2B), suggesting a potential shift toward a pro‐inflammatory tumor microenvironment.

We subsequently established a subcutaneous LLC tumor model to assess the antitumor activity of GA in vivo [26, 28]. We conducted dose‐dependent experiments and found that intratumoral injection of 10 mg/kg of GA in the LLC subcutaneous tumor model led to death in mice. Therefore, we selected doses of 2 mg/kg and 5 mg/kg for comparison of antitumor immunity. Intratumoral injection of GA resulted in a marked, dose‐dependent inhibition of tumor growth, as visually documented (Figure 2C), which was confirmed in serial tumor volume measurements (Figure 2D). Consistent with these observations, tumor weights were significantly reduced in GA‐treated groups compared to controls (Figure 2E), underscoring the robust anti‐tumor efficacy. To delineate the immunologic mechanisms underlying the therapeutic effects of GA, we performed high‐dimensional immune profiling of tumor‐infiltrating cells at the experimental endpoint (see gating strategy, Figure S4A; representative plots, Figure S5A, Supporting information). Compared to the PBS‐treated control group and the group receiving GA at 2 mg/kg, administration of GA at 5 mg/kg significantly enhanced the frequency of mature DCs (MHC‐II^+^CD86^+^ DCs) and neutrophils (Ly6G^+^Ly6C^−^) within the TIME (Figure 2F,G). Furthermore, while GA at 2 mg/kg did not markedly alter macrophage polarization relative to controls, the 5 mg/kg dose promoted a shift toward a pro‐inflammatory M1 phenotype, resulting in an approximately twofold increase in M1 macrophages and a nearly 25% reduction in M2 macrophages (Figure 2H,I). In addition, both doses of GA (2 and 5 mg/kg) led to a substantial increase in tumor‐infiltrating CD8^+^ T cells (Figure 2J), increased IFN‐γ^+^ cells, and reduced FoxP3^+^ regulatory T cells in the 5 mg/kg group compared with the control group. However, the proportion of IFN‐γ^+^ cells and the proportion of FoxP3^+^ regulatory T cells were not significantly changed in the 2 mg/kg group (Figure 2K,L).

We next evaluated GA using an experimental lung metastasis model. Dose‐ranging studies of GA confirmed that intraperitoneal administration of up to 10 mg/kg was well‐tolerated without causing mortality in mice. Accordingly, doses of 2, 5, and 10 mg/kg were selected to evaluate anti‐tumor immunity. Macroscopic examination and histopathological analysis by H&E staining revealed that GA treatment significantly suppressed pulmonary metastasis in a dose‐dependent manner (Figure 2M–O). Consistent with these findings, lung weights were reduced (Figure 2N), indicating a decreased metastatic burden. Immune profiling of lung tissues demonstrated that GA enhanced anti‐tumor immunity in a concentration‐dependent fashion. Specifically, higher doses of GA led to significantly increased infiltration of activated DCs (Figure 2P),Ly6G^+^Ly6C^−^ neutrophils (Figure 2Q), M1 macrophages (Figure 2R), and CD8^+^ T cells (Figure 2S). Furthermore, GA augmented the proportion of IFN‐γ^+^ CD8^+^ T cells (Figure 2T), while dose‐dependently reducing the frequency of immunosuppressive FoxP3^+^ Tregs (Figure 2U; gating strategy shown in Figure S4B, representative plots in Figure S5B, Supporting Information). These results stand in clear contrast to those from the subcutaneous LLC model, where intratumoral injection of 2 mg/kg GA failed to elicit robust anti‐tumor immunity. The differential effects likely stem from distinct pharmacokinetics and drug exposure profiles associated with administration routes: intraperitoneal injection promotes systemic distribution and sustained immune activation, whereas local intratumoral injection may lead to inadequate drug exposure and accelerated clearance, thereby diminishing immunostimulatory efficacy of GA.

We next evaluated the safety profile of intraperitoneal administration of GA in vivo. Hematological and biochemical analyses indicated that while 5 mg/kg and 2 mg/kg GA were well‐tolerated, high‐dose GA administration led to elevated levels of alanine transaminase (ALT) and aspartate transaminase (AST) (Figure S6C, Supporting information), suggesting potential hepatotoxicity. Histopathological examination revealed mild hepatic structural alterations in the high‐dose group (Figure S6D, Supporting information). No significant abnormalities were observed in renal function markers, complete blood counts, or other organ architectures in either the 5 mg/kg or 2 mg/kg group (Figure S6A,B,D, Supporting information). Collectively, these findings demonstrate that GA not only exerts direct antitumor and anti‐metastatic effects but also profoundly remodels the immune landscape toward a pro‐inflammatory phenotype. However, dose‐dependent hepatotoxicity warrants caution for clinical translation, suggesting that mid‐ or low‐dose regimens may offer a more favorable efficacy‐safety profile.

### 2.4 Transcriptomic and Immunohistochemical Analyses Reveal GA‐Induced Pyroptosis Signaling and Immune Checkpoint Adaptation

**FIGURE 3:**
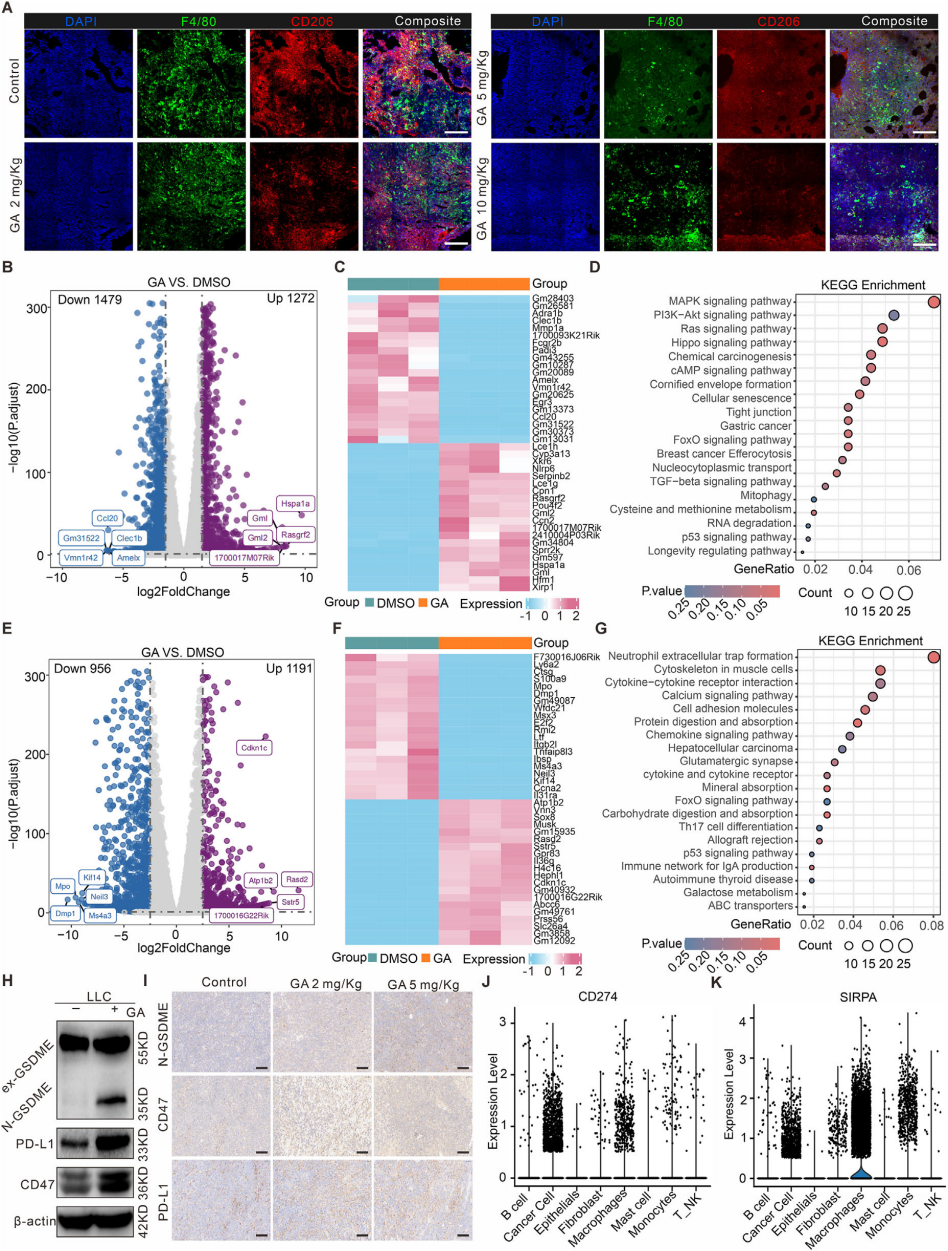
Identification of molecular signaling pathway events related to GA‐induced pyroptosis in LLC cells and reprogramming of M2 macrophages. (A) Assess the expression of CD206 in macrophages within the TIME of LLC lung metastasis models treated with different concentrations of GA by using immunofluorescence. The scale bar is 100 µm. (B‐D) Transcriptomic analysis to identify changes in signaling pathways after treatment of LLC with 10 µmol GA compared to the control group, including volcano plots of differentially expressed genes (B), heatmaps of differentially expressed genes (C), and KEGG pathway enrichment maps related to differentially expressed genes (D). n = 3 biologically independent samples. (E‐G) Transcriptomic analysis to identify changes in signaling pathways after treatment of M2 macrophage with 10 µmol GA compared to the control group, including volcano plots of differentially expressed genes (E), heatmaps of differentially expressed genes (F), and KEGG pathway enrichment maps related to differentially expressed genes (G). n = 3 biologically independent samples. (H) Western blotting was used to evaluate the effects of GA treatment on the expression of PD‐L1 and CD47 in LLC cells. (I) Assess the expression levels of N‐GSDME, PD‐L1, and CD47 in lung tissues of LLC lung metastasis models treated with different concentrations of GA by using immunohistochemistry. The scale bar is 50 µm. (J) Analyze the expression of CD274 (PD‐L1) in different cell subtypes using the online TCGA lung cancer patient database. (K) Analyze the expression of SIRPɑ in different cell subtypes using the online TCGA lung cancer patient database.

To further elucidate the mechanisms underlying GA‐induced pyroptosis and its impact on the TIME, we performed a multi‐level analysis combining immunohistochemistry, transcriptomic profiling, and public database mining. Immunofluorescence staining of lung metastases from GA‐treated mice revealed a significant reduction in CD206^+^ M2 macrophages with increasing GA concentration (Figure 3A), confirming its ability to repolarize macrophages toward an anti‐tumor phenotype i*n vivo*. We next conducted RNA sequencing on GA‐treated LLC cells to delineate the global transcriptional changes associated with GA‐induced pyroptosis. Volcano plots and heatmaps illustrated numerous differentially expressed genes following GA exposure (Figure 3B,C). KEGG pathway enrichment analysis highlighted significant upregulation of genes involved in the p53, MAPK, and FoxO signaling pathways (Figure 3D), all of which are known regulators of caspase‐3 activation and programmed cell death. These findings suggest that GA triggers pyroptosis through convergent stress‐response pathways that ultimately facilitate GSDME cleavage.

Parallel transcriptomic analysis in M2‐polarized macrophages treated with GA revealed profound reprogramming toward an M1‐like phenotype (Figure 3E,F). KEGG enrichment showed prominent activation of cytokine‐cytokine receptor interactions, chemokine signaling, FoxO signaling, Th17 differentiation, and allograft rejection pathways (Figure 3G), consistent with enhanced pro‐inflammatory signaling and immune activation. These changes align with the observed repolarization effects and support the role of GA in remodeling the immunosuppressive microenvironment. Despite these beneficial effects, Western blotting analysis of LLC cells and immunohistochemical staining of lung metastatic tissues indicated that GA treatment led to the upregulation of immune checkpoint molecules, including PD‐L1 and CD47 (Figure 3H,I), which may facilitate immune escape. Interrogation of the TCGA lung cancer database further revealed that CD274 (encoding PD‐L1) was highly expressed not only in tumor cells but also in macrophages from GSDME‐high patients (Figure 3I). Similarly, SIRPα, the cognate receptor for CD47, was elevated in multiple immune subsets (Figure 3J), suggesting a feedback mechanism by which tumor cells counteract pyroptosis‐induced immune activation.

Collectively, these results demonstrate that while GA effectively induces pyroptosis and reprograms macrophage polarization, it concurrently elicits adaptive immunosuppression through upregulation of PD‐L1 and CD47–SIRPα checkpoints. These insights underscore the necessity of combining GA with immune checkpoint blockade to achieve sustained antitumor immunity and provide a rationale for the development of dual‐targeting nanovesicle strategies aimed at disrupting these inhibitory axes.

### 2.5 Engineering and Characterization of Dual‐Targeting Microparticles for Enhanced Delivery of GA

**FIGURE 4:**
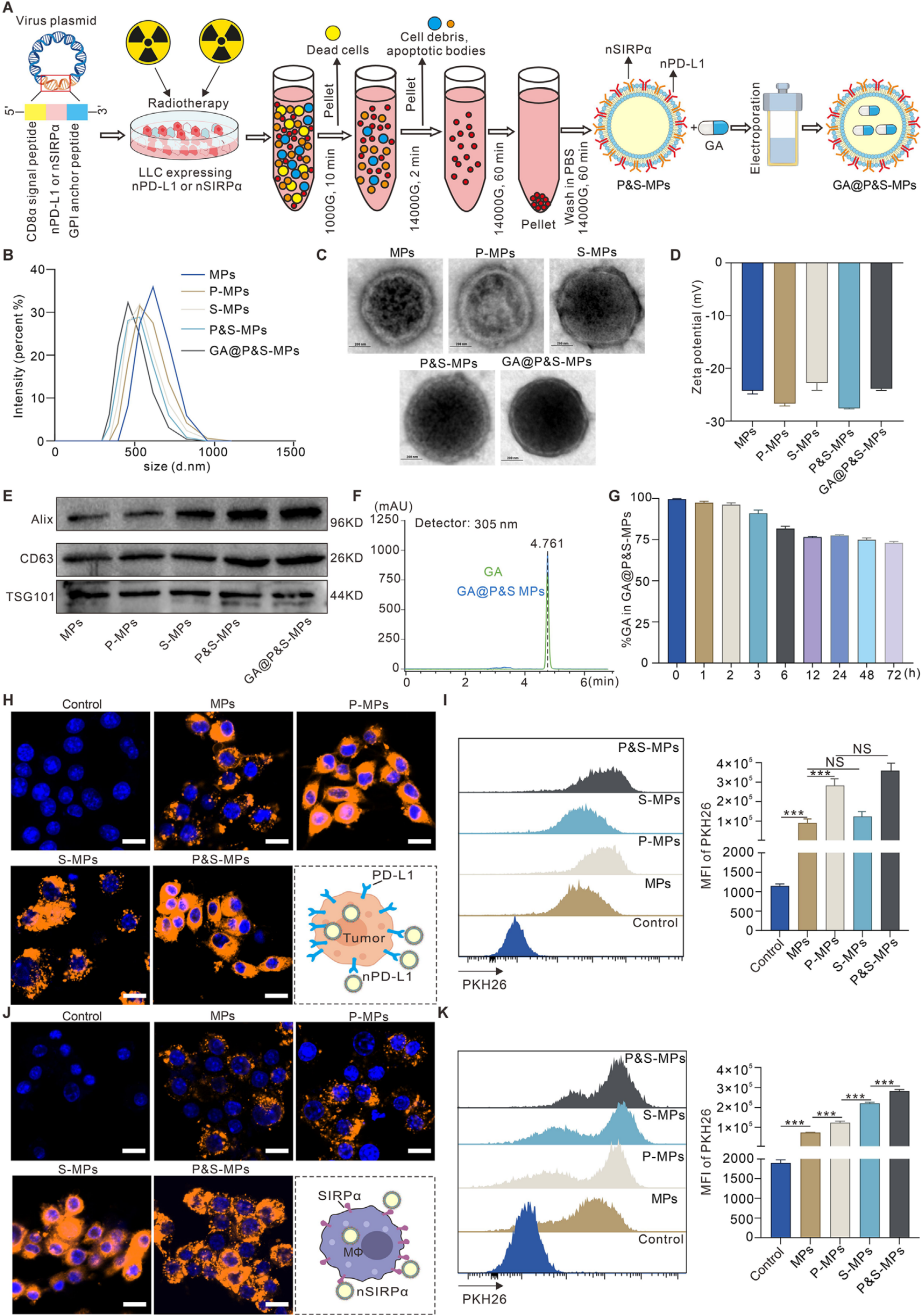
Preparation and characterization of GA@P&S‐MPs. (A) Schematic of GA@P&S‐MPs preparation, containing plasmid construction, MPs purification and GA loading strategy. (B) Size distribution of different engineered MPs measured by dynamic light scattering. (C) Size distribution of different engineered MPs measured by transmission electron microscopy. The scale bar is 200 nm. (D) Zeta potential of different engineered MPs measured by Malvern laser particle size analyzer. n = 3 biologically independent samples. (E) Analysis of the expression of Alix, CD81 and CD63 in different engineered MPs by using western blotting experiment. (F) HPLC to be used to identify the GA loading efficacy of GA@P&S‐MPs. (G) HPLC to be used to identify the drug release characteristics of GA@P&S‐MPs. n = 3 biologically independent samples. (H) Representative images of different engineered MPs uptake in LLC tumor cells obtained by confocal imaging. Nuclei were labeled using DAPI (blue), MPs were pre‐labeled using PKH26 dye (red). The scale bar is 20 µm. (I) Analysis and statistics of the targeting properties of different engineered MPs on LLC tumor cells by flow cytometry. n = 3 biologically independent samples. (J) Representative images of different engineered MPs uptake in RAW264.7 cells obtained by confocal imaging. Nuclei were labeled using DAPI (blue), MPs were pre‐labeled using PKH26 dye (red). The scale bar is 20 µm. (K) Analysis and statistics of the targeting properties of different engineered MPs on RAW264.7 cells by flow cytometry. n = 3 biologically independent samples. Data are presented as mean ± SEM, and the statistical significance was analyzed via one‐way ANOVA. *p < 0.05, **p < 0.01, ***p < 0.001, and NS: not significant.

Motivated by the need to overcome adaptive immune checkpoint upregulation induced by GA monotherapy, we developed a biomimetic delivery system using radiation‐induced MPs. Radiation‐derived MPs were selected based on their previously reported dual capacity to induce ICD and promote macrophage repolarization, coupled with their high drug‐loading efficiency and suitability for genetic engineering [20, 29]. To simultaneously block PD‐L1 and CD47–SIRPα immune inhibitory axes, we engineered LLC cells to express surface‐anchored nanobodies against PD‐L1 [30]and SIRPα [31] via N‐terminal signal peptide and C‐terminal GPI cell membrane‐anchoring sequences. As illustrated in Figure 4A, stably transfected LLC cell lines—expressing either anti‐PD‐L1 nanobody, anti‐SIRPα nanobody, or both—were irradiated to generate functionalized MPs (P‐MPs represents MPs derived from LLC cell expressing anti‐PD‐L1 nanobody, S‐MPs represents MPs derived from LLC cell expressing anti‐SIRPα nanobody and P&S‐MPs represents MPs derived from LLC cell expressing anti‐SIRPα nanobody and anti‐PD‐L1 nanobody), which were subsequently loaded with GA via electroporation, yielding the final product GA@P&S‐MPs.

We first characterized the physical properties of the engineered MPs. Dynamic light scattering (DLS) and transmission electron microscopy (TEM) revealed uniform size distributions across all MP types, with diameters ranging between 600–900 nm (Figure 4B,C). Zeta potential measurements confirmed moderate negative surface charges, indicative of good colloidal stability (Figure 4D). Western blot analysis confirmed the presence of MPs markers (Alix, CD81, CD63) in all MPs formulations, affirming their vesicular identity (Figure 4E). In addition, Western blot analysis and flow cytometry further confirmed that S‐MPs, P‐MPs, and P&S‐MPs carried the corresponding nanobodies, respectively (Figure S7A,B, Supporting Information). Drug loading and release profiles were critically evaluated. High‐performance liquid chromatography (HPLC) demonstrated that GA was efficiently encapsulated into P&S‐MPs with a loading efficiency of approximately 78% (Figure 4F). In vitro release kinetics under physiological conditions showed sustained GA release over 48 h, with nearly 80% cumulative release, suggesting the potential for prolonged action (Figure 4G).

We next assessed the cellular targeting specificity of the engineered MPs. Confocal microscopy and flow cytometry in LLC tumor cells demonstrated significantly enhanced uptake of P&S‐MPs compared to non‐targeted MPs or single‐ligand MPs, as evidenced by PKH26 red fluorescence (Figure 4H,I). Similarly, in RAW264.7 macrophages, P&S‐MPs exhibited superior binding and internalization (Figure 4J,K), underscoring their dual‐targeting capability toward both tumor cells and immune cells. Collectively, these results establish GA@P&S‐MPs as a robust, genetically engineered delivery system that combines efficient GA loading with specific targeting of PD‐L1 and SIRPα. This approach not only enhances drug delivery but also concurrently neutralizes key immune evasion mechanisms, providing a promising combinatorial strategy for augmenting antitumor immunity.

### 2.6 Functional Validation of GA@P&S‐MPs

**FIGURE 5:**
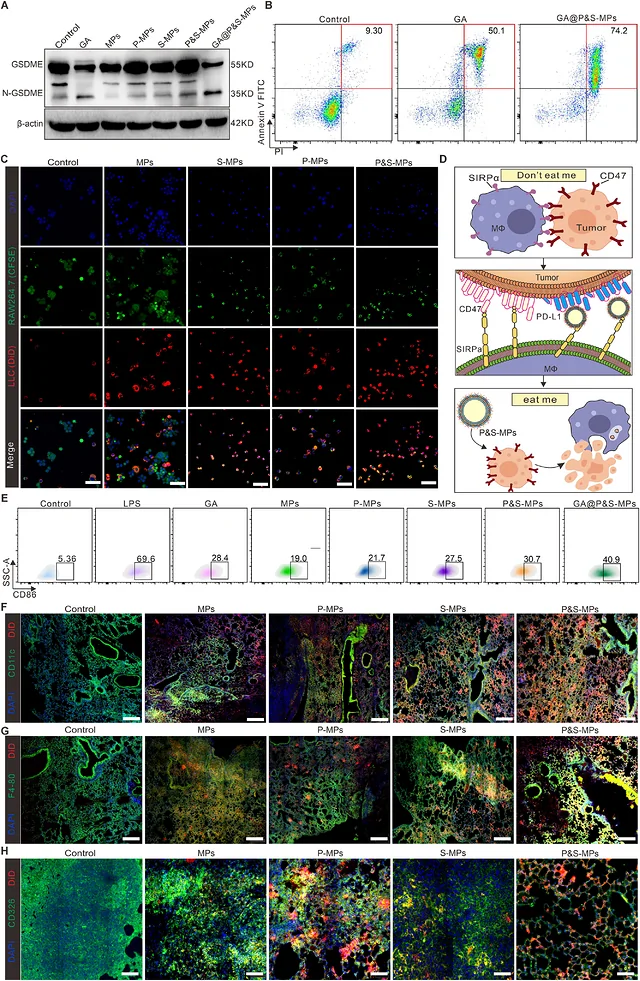
Characterization of the functional properties of GA@P&S‐MPs and evaluation of their TIME targeting characteristics in vivo. (A) Evaluate the ability of different engineered MPs to induce GSDME cleavage in LLC cells in vitro by using western blotting experiment. (B) Assess the consistency in the mode of cell death induced by GA and GA@P&S‐MPs in LLC cells. n = 3 biologically independent samples. (C) Representative confocal imaging of the phagocytic effect of RAW264.7 cells incubated with LLC cells pretreated with different engineered MPs. The scale bar is 50 µm. (D) Schematic diagram showing the role of nPD‐L1 and nSIRPɑ in P&S‐MPs as bispecific adapters to promote contact between macrophages and tumor cells and simultaneously blocking the CD47/SIRPɑ signal. (E) Flow cytometry was used to evaluate the ability of different engineered MPs to induce reprogramming of M2 macrophages. n = 3 biologically independent samples. (F) Targeting characteristics of different engineered MPs on DCs in lung metastasis models by using immunofluorescence. The scale bar is 100 µm. (G) Assess the targeting characteristics of different engineered MPs on macrophages in lung metastasis models analyzed by immunofluorescence. The scale bar is 100 µm. (H) Targeting characteristics of different engineered MPs on tumor cells (CD326 positive) in lung metastasis models analyzed by immunofluorescence. The scale bar is 100 µm.

We next evaluated the therapeutic efficacy of GA@P&S‐MPs in inducing pyroptosis and modulating tumor‐immune interactions. Western blot analysis confirmed that GA@P&S‐MPs effectively induced cleavage of GSDME in LLC cells, with efficiency comparable to—or greater than—that of free GA at an equivalent concentration (10 µM) (Figure 5A). Consistent with these findings, CCK‐8 assays revealed significant reduction in cell viability following treatment with GA@P&S‐MPs (Figure S7C, Supporting information). Flow cytometric analysis further demonstrated that GA@P&S‐MPs induced high levels of apoptosis, as indicated by high Annexin V^+^/PI^+^ populations (Figure 5B and Figure S7D, Supporting information). Moreover, GA@P&S‐MPs triggered substantial LDH release, exceeding the levels observed with free GA (Figure S7E, Supporting information), confirming enhanced pyroptotic cell death. We next investigated whether engineered MPs could serve as bispecific adapters to facilitate macrophage‐tumor cell interactions independent of GA. Using a co‐culture system of RAW264.7 macrophages and DiD‐labeled LLC cells, confocal microscopy revealed that untreated MPs did not promote phagocytosis. However, P‐MPs (anti‐PD‐L1) and S‐MPs (anti‐SIRPα) moderately enhanced macrophage engulfment of tumor cells, with the greatest effect observed in the dual‐targeted P&S‐MPs group, where nearly all macrophages contained internalized LLC cells (Figure 5C). These observations were quantitatively supported by flow cytometry (Figure S7F, Supporting information). In Figure 5D, we show a proposed mechanism for the role of P&S‐MPs as bispecific adapters, simultaneously binding tumor cells and macrophages via nanobody engagement while blocking the CD47‐SIRPα “don't eat me” signal, thereby drastically enhancing phagocytic efficiency (Figure 5D).

Beyond promoting phagocytosis, GA@P&S‐MPs also potently reprogrammed M2‐polarized macrophages toward an M1‐like phenotype, as evidenced by significantly elevated CD86 expression in BMDMs (Figure 5E and Figure S7G, Supporting information). Finally, we assessed the in vivo targeting specificity of engineered MPs within the lung metastatic microenvironment. Immunofluorescence imaging and flow cytometric analysis demonstrated that P&S‐MPs efficiently accumulated in DCs, macrophages, and tumor cells (CD326^+^) in lung tissues (Figure 5F–H and Figure S7H–K, Supporting information). Notably, dual‐functionalized MPs exhibited superior targeting compared to single‐ligand formulations, highlighting their enhanced ability to specifically deliver cargo to both immune and tumor compartments. Together, these results establish that GA@P&S‐MPs not only retain the pro‐pyroptotic activity of GA but also confer additional immunomodulatory functions—including enhanced phagocytosis and macrophage reprogramming—through their engineered surface nanobodies. Moreover, their specific targeting to key cellular components of the TIME supports their potential as a precision combinatorial therapy for lung cancer.

### 2.7 GA@P&S‐MPs Exhibit Superior Antitumor Efficacy and Immune Microenvironment Remodeling With Reduced Toxicity Compared to Monotherapy

**FIGURE 6:**
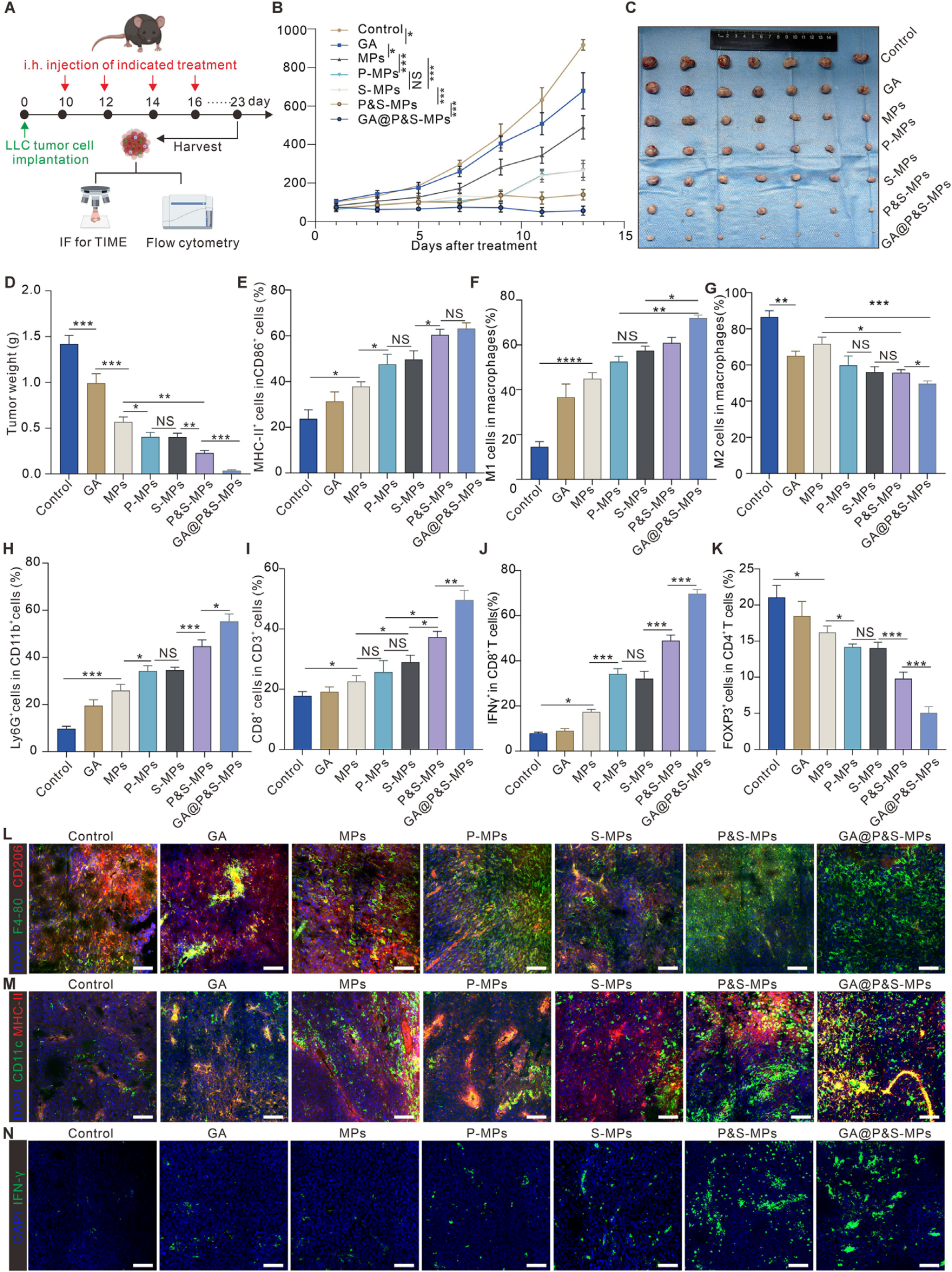
Validation of the therapeutic mechanisms of indicated engineered MPs for treating LLC subcutaneous tumors in vivo. (A) Schematic diagram depicting the analysis of engineered MPs in the therapy of an LLC subcutaneous tumors model. (B) Statistical analysis of tumor size in mice treated with the indicated engineered MPs. n = 7 mice per group. (C) In vitro tumor size in LLC‐tumor‐cell‐bearing mice treated with engineered MPs. (D) Characterization of the effects of GA on the weight of LLC subcutaneous tumors in mice. (E‐K) Effects of engineered MP therapy on the proportion and number of immune cell subsets in the TIME. n = 5 mice per group. (E) Statistical analysis of the proportion of MHC‐II^+^CD86^+^DCs in the total population of tumor‐infiltrating DCs. (F) Statistical analysis of the proportion of M1 macrophages in the total population of tumor‐infiltrating macrophages. (G) Statistical analysis of the proportion of M2 macrophages in the total population of tumor‐infiltrating macrophages. (H) Statistical analysis of the proportion of Ly6G^+^Ly6c^−^ neutrophils in the total population of CD11b^+^ tumor‐infiltrating myeloid cells. (I) Statistical analysis of the proportion of CD8^+^ T cells in the total population of tumor‐infiltrating CD3^+^ T cells. (J) Statistical analysis of the proportion of IFN‐γ^+^ T cells in the total population of tumor‐infiltrating CD8^+^ T cells. (K) Statistical analysis of the proportion of FoxP3^+^ T cells in the total population of tumor‐infiltrating CD8^+^ T cells. (L) The ability of engineered MPs to induce reprogramming in macrophages derived from LLC subcutaneous tumors analyzed via immunofluorescence. The scale bar is 100 µm. (M) The ability of engineered MPs to induce the maturation of DCs in LLC subcutaneous tumors analyzed by immunofluorescence. The scale bar is 100 µm. (N) The ability of engineered MPs to induce the expression of IFN‐γ in LLC subcutaneous tumors analyzed by immunofluorescence. The scale bar is 100 µm. Data are presented as mean ± SEM, and statistical significance was analyzed via one‐way ANOVA. *p < 0.05, **p < 0.01, ***p < 0.001, and NS: not significant.

We sought to determine whether encapsulating GA within dual‐targeting MPs could enhance its therapeutic index. In the subcutaneous LLC model (Figure 6A), GA@P&S‐MPs—administered at the same GA equivalent dose of 2 mg/kg—elicited significantly stronger suppression of subcutaneous tumor growth than free GA and other indicated treatment (Figure 6B). The results demonstrated that, compared to the PBS group, GA monotherapy only reduced tumor volume by 25%. In contrast, treatment with P&S‐MPs led to a nearly 85% reduction in average subcutaneous tumor volume. MPs alone decreased tumor volume by approximately 50%, while either P‐MPs or S‐MPs reduced tumor volume to about 25% of that in the PBS group. These findings reveal that MPs combined with either PD‐L1 or SIRPα targeting can effectively inhibit the growth of LLC subcutaneous tumors. Moreover, simultaneous blockade of both PD‐L1 and SIRPα exhibited a stronger antitumor effect, confirming the synergistic activity of bispecific engagers and MPs in treating LLC. Furthermore, when GA was co‐loaded into P&S‐MPs, tumor volume was reduced to just 6% of that in the PBS group, nearly achieving complete eradication of the tumors. These observations were consistent with the data obtained from excised tumor sizes (Figure 6C) and tumor weights (Figure 6D). This suggests that the engineered delivery system substantially enhances drug bioavailability and tumor targeting. Strikingly, while free GA at 2 mg/kg showed limited immunomodulatory effects in earlier analyses, GA@P&S‐MPs drove profound reprogramming of the TIME (gating strategy in Figure S8A; representative plots in Figure S9, Supporting information). Notably, S‐MPs, which display anti‐SIRPα nanobodies, augmented innate immunity by enhancing activation of DCs (Figure 6E), skewing macrophage polarization from M2 to M1 (Figure 6F,G), and reducing Ly6G^+^Ly6C^−^ neutrophils (Figure 6H). In addition, the inclusion of anti‐PD‐L1 nanobodies on P‐MPs enhanced adaptive immune activation, as evidenced by increased infiltration of CD8^+^ T cells (Figure 6I) and elevated abundance of IFN‐γ^+^ cytotoxic T cells (Figure 6J), while reducing FoxP3^+^ regulatory T cells (Figure 6K). The combination of nanobodies and GA in GA@P&S‐MPs produced synergistic effects, significantly promoting a pro‐inflammatory tumor microenvironment. These results were further supported by immunofluorescence staining, which revealed enhanced macrophage reprogramming, DC maturation, and IFN‐γ expression in tumor tissues (Figure 6L–N).

**FIGURE 7:**
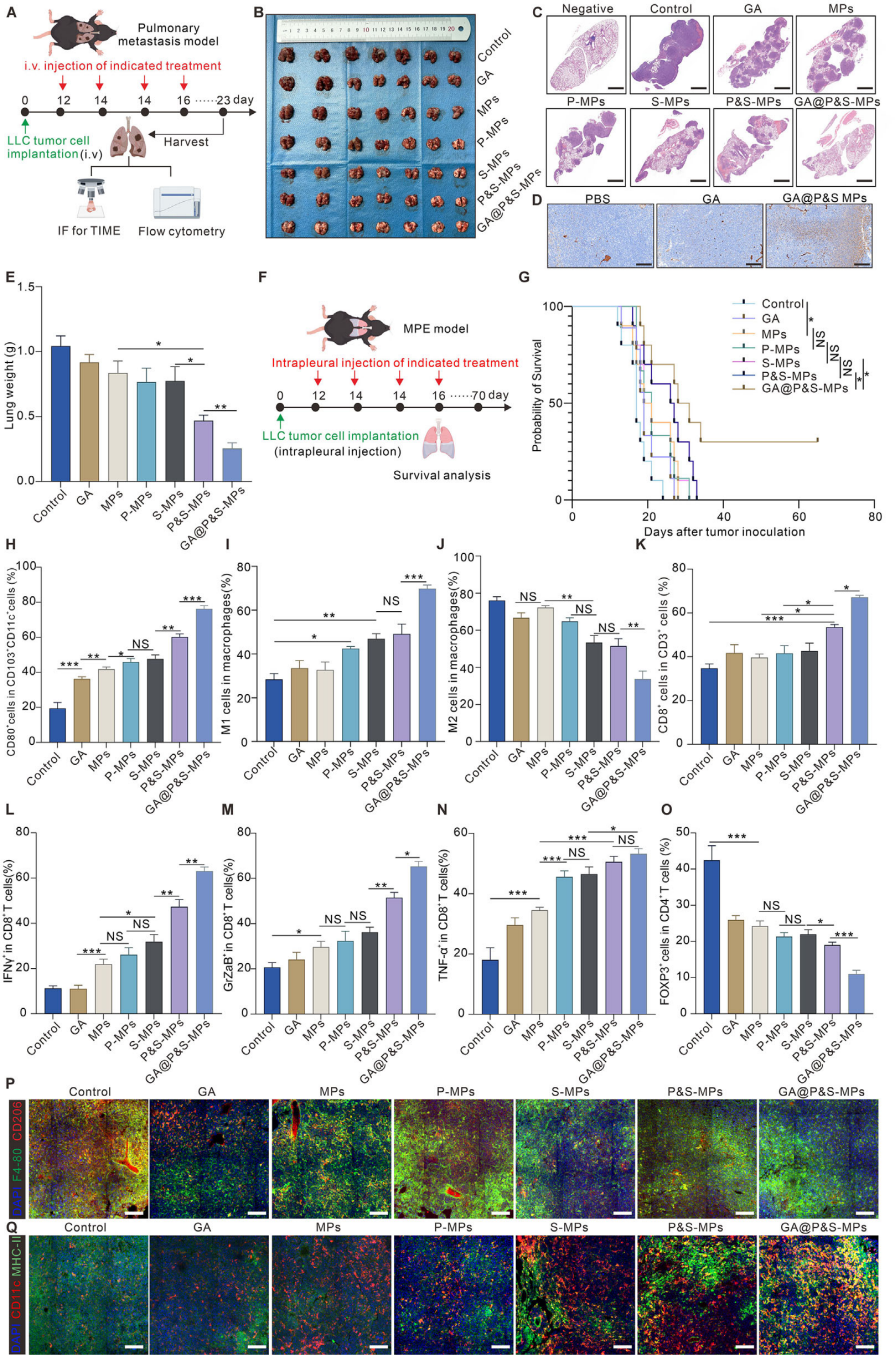
Validation of the therapeutic mechanisms of engineered MPs for treating lung metastasis or MPE in LLC bearing mice in vivo. (A) Schematic diagram illustrating the use of engineered MPs for treatment of a lung metastasis model constructed by subcutaneous injection of LLC tumor cells. (B) Physical images of in vitro lung metastasis in LLC bearing mice treated with engineered MPs. n = 7 mice per group. (C) Pulmonary metastasis of LLC cells in vivo after treatment with indicated engineered MPs using hematoxylin and eosin (HE) staining. The scale bar is 1 mm. (D) Immunohistochemical evaluation of GA and the characteristics of pyroptosis in lung cancer mediated by GA@P&S‐MPs carrying GA. The scale bar is 50 µm. (E) Characterization of the effects of engineered MPs on lung weight in mice. (F) Schematic diagram of the experimental chart for engineered MPs in the treatment of an MPE model constructed by intrapleural injection of LLC tumor cells. (G) Statistical analysis of the survival rate in mice treated with the indicated engineered MPs or GA. n = 10 mice per group. (H‐O) Effects engineered MP therapy on the proportion and number of immune cell subsets in the TIME. n = 5 mice per group. (H) Statistical analysis of the proportion of CD80^+^DCs in the total population of tumor‐infiltrating DCs. (I) Statistical analysis of the proportion of M1 macrophages in the total tumor‐infiltrating macrophage population. (J) Statistical analysis of the proportion of M2 macrophages in the total population of tumor‐infiltrating macrophages. (K) Statistical analysis of the proportion of CD8^+^ T cells in the total population of tumor‐infiltrating CD3^+^ T cells. (L) Statistical analysis of the proportion of IFN‐γ^+^ T cells in the total population of tumor‐infiltrating CD8^+^ T cells. (M) Statistical analysis of the proportion of GzmB^+^ T cells in the total population of tumor‐infiltrating CD8^+^ T cells. (N) Statistical analysis of the proportion of TNF‐ɑ^+^ T cells in the total population of tumor‐infiltrating CD8^+^ T cells. (O) Statistical analysis of the proportion of FoxP3^+^ T cells in the total population of tumor‐infiltrating CD8^+^ T cells. Immunofluorescence analysis of the ability of (P) engineered MPs to induce reprogramming of macrophages in a lung metastasis model; scale: 100 µm and (Q) the ability of engineered MPs to induce the maturation of DCs in a lung metastasis model; scale: 100 µm. Data are presented as mean ± SEM, and statistical significance was analyzed via one‐way ANOVA. *p < 0.05, **p < 0.01, ***p < 0.001, and NS: not significant.

We next extended our investigation to a metastatic lung cancer model (Figure 7A). Macroscopic examination, H&E and Immunohistochemistry (N‐GSDME) staining revealed that GA@P&S‐MPs strongly inhibited lung metastasis formation and mediating robust pyroptosis in situ tumors compared to other groups (Figure 7B–D), accompanied by reduced lung weights (Figure 7E). To further evaluate the therapeutic efficacy of GA@P&S‐MPs in an advanced lung cancer model, we established a malignant pleural effusion (MPE) model using LLC cells, which recapitulates clinical features of end‐stage incurable lung cancer (Figure 7F). Compared to the PBS group, GA treatment alone did not significantly prolong survival in the MPE model. Although MPs, P‐MPs, S‐MPs, and P&S‐MPs all markedly extended mouse survival compared to PBS, no statistically significant differences were observed among these MP‐based treatments alone. This indicates that immunomodulation of the MPE microenvironment by MPs is insufficient to substantially improve survival outcomes. In contrast, GA@P&S‐MPs, which co‐deliver GA, significantly enhanced survival in the MPE model and achieved a 30% complete cure rate, underscoring the critical role of pyroptosis induction in effectively treating MPE (Figure 7G). To verify the changes in the pulmonary immune microenvironment mediated by GA@P&S‐MPs, we conducted further analysis of immune cell infiltration. Immune contexture analysis of lung metastatic tissues (gating strategy shown in Figure S8B; representative plots in Figure S10, Supporting Information) demonstrated that GA@P&S‐MPs increased the abundance of mature DCs (Figure 7H), M1 macrophages (Figure 7I), and CD8^+^ T cells (Figure 7K), while reducing M2 macrophages (Figure 7J). Additionally, GA@P&S‐MPs enhanced the functionality of CD8^+^ T cells, as indicated by elevated proportions of IFN‐γ^+^, GzmB^+^, and TNF‐α^+^ cells (Figure 7L–N), and reduced FoxP3^+^ Treg infiltration (Figure 7O). These findings were visually confirmed via immunofluorescence, which showed enhanced macrophage repolarization and DC maturation in metastatic lung tissues (Figure 7P,Q), supporting the ability of the dual‐targeting system to overcome the limited efficacy of free GA at low dose.

Given the potent efficacy observed at low‐dose GA (2 mg/kg), we further evaluated the safety profile of GA@P&S‐MPs. All blood parameters, liver and kidney function markers, and major organ architectures remained within normal limits (Figure S11A–D, Supporting Information), indicating that the targeted combination strategy significantly improves the therapeutic window of GA by minimizing off‐target effects and maximizing immune activation. In summary, GA@P&S‐MPs exert potent antitumor effects in both localized and disseminated tumor models through synergistic mechanisms: GA‐induced pyroptosis initiates ICD, while PD‐L1 and SIRPα nanobodies jointly overcome adaptive and innate immune suppression. This integrated strategy results in profound and sustained antitumor immunity with a favorable safety profile.

## 3 Discussion

In this study, we established GSDME as a critical mediator of pyroptosis in lung cancer with profound implications for prognosis and immune modulation. We demonstrated that GSDME expression is significantly elevated in lung tumor tissues, and its activation state is closely associated with patient survival and immune cell communication.

We found that GA differs from other chemotherapeutic drugs that induce Caspase‐3 activation and subsequent GSDME cleavage. GA can directly bind to arginine 207 of Caspase‐3, enhancing the interaction between Caspase‐3 and GSDME, thereby effectively inducing GSDME cleavage and pyroptosis execution. GA exhibited robust anti‐tumor effects* in vivo*, inhibiting tumor growth and metastasis and reprogramming the TIME toward a pro‐inflammatory state by promoting M1 macrophage polarization, DCs maturation, and cytotoxic T cell infiltration. However, we also observed that GA treatment led to adaptive upregulation of immune checkpoints PD‐L1 and CD47, suggesting a feedback mechanism that may limit long‐term efficacy.

To address this limitation, we developed a dual‐targeting MP system, GA@P&S‐MPs, which co‐delivers GA and nanobodies against PD‐L1 and SIRPα using MPs derived from irradiated tumor cells. Our dual‐targeting nanobody design offers key advantages over single‐targeting agents. It synergistically blocks both the PD‐1/PD‐L1 and CD47/SIRPα pathways, more effectively disrupting the TIME [32, 33]. Simultaneous engagement of PD‐L1 on tumor cells and SIRPα on macrophages enhances tumor‐specific accumulation, reduces off‐target effects, and overcomes antigen heterogeneity. This strategy amplifies GA‐induced pyroptosis and bridges innate with adaptive immunity, as evidenced by improved T‐cell responses and macrophage reprogramming in vivo, presenting a promising approach for treating immunologically “cold” tumors. These MPs are particularly suitable as immunomodulatory nanocarriers, as they inherently mimic the effects of radiotherapy and carry radiation‐induced DAMPs, which enhance immunogenic signaling and promote dendritic cell maturation. This property allows the vesicles to not only serve as a delivery vehicle but also to recapitulate aspects of the abscopal effect, thereby priming the tumor microenvironment for enhanced anti‐tumor immunity. Consequently, the GA@P&S‐MPs system not only enhanced pyroptosis induction and macrophage phagocytosis but also effectively blocked key immune inhibitory pathways. The combination of GA‐induced ICD and dual checkpoint inhibition resulted in synergistic anti‐tumor immunity, significantly improving survival in metastatic and effusion models without evident systemic toxicity. Our work underscores the potential of leveraging pyroptosis to activate anti‐tumor immunity while simultaneously counteracting adaptive immune resistance. The GA@P&S‐MPs platform represents a promising nanotherapeutic strategy for lung cancer, particularly for tumors with high GSDME expression. Given the ability of GA@P&S‐MPs to induce robust pyroptosis and remodel the immune microenvironment, this approach holds significant promise for the treatment of immunologically “cold” tumors, which are typically resistant to conventional ICB therapy. By simultaneously promoting ICD and blocking key immune inhibitory pathways, GA@P&S‐MPs may effectively convert cold tumors into immunologically hot tumors, facilitating enhanced T cell infiltration and sustained anti‐tumor responses. Future studies should explore the applicability of this approach in other cancer types and evaluate its combination with other immunomodulatory agents to achieve sustained and broad anti‐tumor responses.

## 4 Methods

### 4.1 Data Acquisition

We obtained single‐cell RNA sequencing (scRNA‐seq) data from the Gene Expression Omnibus (GEO) (https://www.ncbi.nlm.nih.gov/) database (accession number: GSE148071), which comprised a total of 42 samples and 89 819 cells for subsequent analysis. Bulk RNA‐seq data across 33 cancer types were downloaded from the TCGA database via the XENA platform (https://xena.ucsc.edu/), along with normal tissue transcriptomic data from the GTEx project, which served as non‐cancerous controls for comparative analyses.

### 4.2 Quality Control

We performed standard quality control on the raw single‐cell expression data using the subset function in Seurat. Cells were retained only if they expressed more than 200 and fewer than 4000 genes (nFeature_RNA), contained less than 15 000 total UMIs (nCount_RNA), and had mitochondrial gene percentages below 20% (percent.mt). These thresholds were chosen to remove low‐quality cells, empty droplets, and potential doublets while preserving biologically meaningful cell populations. Following this filtering step, the dataset was reduced from 89 819 cells to 55 131 high‐quality cells for subsequent analysis.

### 4.3 Preprocessing of scRNA‐seq data

Following quality control, the single‐cell expression data were normalized using the NormalizeData function with default parameters to adjust for sequencing depth. Subsequently, 3000 highly variable genes were identified using FindVariableFeatures to focus downstream analyses on genes with strong biological signals. All genes were scaled using ScaleData to regress out unwanted sources of variation and facilitate dimensional reduction. Principal component analysis (PCA) was performed to reduce dimensionality, and the number of significant principal components (PCs = 25) was determined based on the elbow plot method via ElbowPlot. To mitigate potential batch effects, harmony integration was applied using RunHarmony. A shared nearest‐neighbor graph was constructed with FindNeighbors using the harmonized PCs, and cell clusters were identified with FindClusters, which implements a modularity optimization‐based algorithm. Finally, non‐linear dimensional reduction was carried out with RunUMAP to visualize the cell clusters in two dimensions.

### 4.4 Cell Type Identification of scRNA‐seq Data

We annotated each cell cluster according to well‐known cell markers provided by original literatures, resulting in a total of seven major cell types, including T_NK cell (CD3D, CD3E, NKG7, KLRD1), B cells (CD79A, MZB1), mast cells (TPSAB1, CPA3), macrophages (CD163, MARCO, MRC1), monocytes (FCN1, IL1B), fibroblasts (DCN, COL1A1) and epithelial cells (EPCAM, KRT18) identified in epithelial cells; epithelial_1 (EGFR, MYC, SOX2) and epithelial_2 (CAPS, SNTN).

### 4.5 Identifying Malignant Cells

To distinguish malignant from non‐malignant cells, we performed copy number variation (CNV) inference analysis using infercnv, a computational tool designed to detect large‐scale chromosomal alterations in single‐cell RNA‐seq data. T cells, NK cells and B cells were selected as reference cells due to their stable genomic profiles. By comparing the CNV patterns of epithelial cells against these reference populations, we identified epithelial_1 having significantly higher CNV burdens, indicative of malignancy.

### 4.6 Cell to Cell Communication

To infer cell‐cell communication networks from the single‐cell transcriptomic data, we applied the CellChat package integrated with ligand‐receptor interaction information from CellPhoneDB. This approach leverages a curated database of molecular interactions to systematically identify and quantify signaling probabilities between cell clusters, enabling the identification of key outgoing and incoming signaling pathways across distinct cell types. The resulting communication networks were further analyzed to uncover biologically relevant interactions and visualized to highlight central signaling mechanisms.

### 4.7 Differential Gene Expression Analysis

Differential gene expression analysis was performed using two distinct statistical approaches tailored to the respective data types and research questions. For the analysis of TCGA data, we conducted gene‐wise differential expression testing using the non‐parametric Wilcoxon rank‐sum test. This approach was employed to compare expression levels of pre‐specified candidate genes between tumor and normal adjacent tissue samples. For the bulk RNA‐seq data obtained from LLC cells and macrophage samples, we performed comprehensive differential expression analysis using DESeq2, which utilizes a negative binomial generalized linear model to account for over‐dispersion in read count data. Genes were considered significantly differentially expressed if |log2 Fold Change| > 1.5 (LLC) or |log2 Fold Change| > 2.5 (macrophage) and *p* adjust < 0.05 (Benjamini‐Hochberg procedure).

### 4.8 Survival Analysis

The optimal cutoff value for stratifying patients into high and low expression groups for each gene was determined by using the surv_cutpoint function from the R package survminer. Based on this gene‐specific cutoff, samples were categorized accordingly. Kaplan‐Meier survival curves were then generated via the survival package to visualize the association between gene expression levels and overall survival. The statistical significance of the observed survival differences between groups was assessed using the log‐rank test.

### 4.9 KEGG Enrich Analysis

To identify biologically relevant pathways associated with the upregulated differentially expressed genes, we performed KEGG pathway enrichment analysis using the clusterProfiler R package. A hypergeometric test was applied to evaluate the statistical representation of the gene set in each KEGG pathway, with a significance threshold set at *p* value < 0.05. The results were visualized using dot plots to display enriched pathways based on gene ratio and *p* value. Special emphasis was placed on pathways related to cell death, immune regulation, and cancer to interpret their potential biological implications.

### 4.10 Cell Lines

Lewis, MC38, 4T1, GL261, H22, and CT26 cell lines were purchased from Procell Life Science & Technology Co., Ltd. HEK293T, and RAW264.7 cell lines were kindly provided by Union Hospital, Tongji Medical College, Huazhong University of Science and Technology. H22 and CT26 cells were cultured in RPMI 1640 medium (Gibco, Grand Island, NY, USA) containing 10% fetal bovine serum. 293T, Lewis, MC38, 4T1, GL261, RAW264.7 cell lines were cultured in Dulbecco's Modified Eagle's Medium (DMEM) (Gibco, Grand Island, NY, USA) containing 10% fetal bovine serum. The cells were cultured at 37°C cell incubator containing 5% carbon dioxide.

### 4.11 Plasmids

We constructed plasmids for GSDMD, GSDME, and caspase3. Mouse GSDMD, GSDME, and caspase3 were cloned into a phage vector using cDNA from the LLC cell line as the template (the vector was provided by Prof. Duan Qiuhong from the Basic Medical School of Huazhong University of Science and Technology). The caspase3 mutant was generated using the DpnI mutagenesis kit (Thermo) and verified by sequencing. The plasmid PLVX‐CD8α‐PD‐L1Nb‐GPI‐G418 and PCDH‐CD8α‐SIPRαNb‐GPI‐Puro were purchased from Beijing Qingke Biotechnology Co., Ltd. All amino acid sequence and primer sequence used in this research are shown in Table S1.

### 4.12 Western Blotting and Antibodies

The cell lysates were separated by SDS‐PHAGE and transferred to PVDF membrane. The PVDF membranes were blocked with 5% skimmed milk powder, then washed with PBST buffer, and incubated with the corresponding antibodies overnight at 4°C. Next membranes were cleaned with PBST buffer, and incubated with the corresponding secondary antibodies at room temperature for 1 h. Membranes were immersed in the developer and developed by Bio‐Rad ChemiDoc XRS. The antibodies used were as follows: GSDME (Abcam; ab215191), GSDMD (Abcam; ab209845), caspase3 (Cell signaling Technology; 9662), cleaved‐caspase3 (Cell signaling Technology; 9661), β‐actin (SANTA; 47778), PD‐L1 (proteintech; 28076‐1‐AP), CD47 (proteintech;85958‐1‐RR). Alix (proteintech; 12422‐1‐AP), CD63 (proteintech; 32151‐1‐AP), and TSG101 (proteintech; 28283‐1‐AP).

### 4.13 Drug Screening

Pyroptosis Compound Library was purchased from MCE (HY‐L059), Cells were seeded at 4000 cells per well in 96‐well plates. Cell viability was assessed using the Cell Counting Kit 8 (biosharp; BS350A) assay 24 h after drug treatment. Following this, the cells were subjected to staining with Hoechst 33342 /PI (Biosharp; BL116A) to assess cell viability and perform screening.

### 4.14 Drug Treatment

LLC cells were seeded into 6‐cm dishes 24 h before stimulation. The cells were treated with Geldanamycin (MCE; HY‐15230) for 24 h, or pretreated with ZVAD (MCE; HY‐164388) or DEVD (MCE; HY‐12466) for 2 h followed by Geldanamycin treatment for 24 h. Subsequently, proteins were harvested for western blot analysis.

### 4.15 Live‐Cell Imaging

Lewis lung carcinoma (LLC) cells were seeded into confocal dishes at a density of 5 × 10^4^ cells per well. After cell adhesion, the medium was replaced with fresh complete medium containing GA, followed by immediate addition of propidium iodide (PI) at a final concentration of 1 µg/mL to label cells with compromised membrane integrity. The dish was then placed on the stage of a confocal microscope equipped with an environmental control chamber (maintained at 37°C and 5% CO_2_). Imaging was performed using a 20× objective. The excitation/emission wavelengths for PI were set, and bright‐field images were acquired simultaneously. Immediately after drug addition, timing was initiated, and images from multiple fields of view were continuously captured.

### 4.16 LDH Release Assay

Pyroptosis was quantified by measuring LDH activity in the cell culture supernatant using an LDH release assay kit (Beyotime, C0016), in accordance with the manufacturer's instructions. Following treatments and transfections, the amount of LDH released was measured and expressed as a percentage of the total LDH activity present in the corresponding cell lysate.

### 4.17 Analysis of Cell Apoptosis

Cells were cultured in 6 well plates (30 000 cells per well) and then incubated with PBS (control), GA, and 20 µg mL^−1^ GA@P&S‐MPs for 6 h. Apoptosis was evaluated by Annexin V‐Alexa Fluor 488/PI apoptosis detection kit (absin, abs50001).

### 4.18 Prediction of Potential Candidate Targets for Geldanamycin

The potential targets of geldanamycin were predicted using the PharmMapper server [32]. Briefly, the 3D structure of geldanamycin in SDF format was retrieved from the PubChem database (https://pubchem.ncbi.nlm.nih.gov/). This file was then uploaded to the PharmMapper online platform (http://www.lilab‐ecust.cn/pharmmapper/). The following parameter settings were applied: Generate Conformers: Yes; Maximum Generated Conformations: 300; Select Targets Set: Human Protein Targets only; Number of Reserved Matched Targets: 300. The Fit score and z'‐score provided in the output were used as key criteria for evaluating potential target proteins. Based on the Fit score, caspase 3 was identified as a candidate target of geldanamycin.

### 4.19 Molecular Docking

Molecular docking was performed using AutoDock to investigate the binding interactions between geldanamycin and caspase3. The protein structure (PDB ID:2H5I) was prepared by removing water molecules and adding hydrogen atoms. The ligand was energy‐minimized and prepared for docking using AutoDock tools. A grid box was centered on the binding site with dimensions of [X] × [Y] × [Z] Å. Docking calculations were carried out using the Lamarckian Genetic Algorithm with 100 runs per ligand. The resulting poses were analyzed based on binding energy and cluster analysis. The docking results were visualized using PyMOL to examine the binding mode and molecular interactions between the ligand and protein. The pose with the lowest binding energy was selected for further analysis.

### 4.20 Molecular Dynamics Simulation

All molecular dynamics (MD) simulations were performed using GROMACS 2022. The receptor protein was described with the AMBER14SB force field, while the ligand parameters were derived from the GAFF2 force field using the sobtop_1.0(dev3.1) software, with atomic charges assigned via the RESP method. The system was solvated in a cubic box of TIP3P water with a 1.0 nm extension from the solute and neutralized with 0.15 M NaCl. Long‐range electrostatics were treated using the Particle Mesh Ewald (PME) method with a 1.0 nm cutoff. Bonds were constrained using the LINCS algorithm.

The system was energy‐minimized using a combination of steepest descent and conjugate gradient algorithms. Subsequently, it was equilibrated under NVT and NPT ensembles. Production MD simulations were conducted for 100 ns under NPT conditions (310 K, 1 bar) using the Nosé–Hoover thermostat and Parrinello–Rahman barostat, with an integration time step of 2 fs. Trajectory analysis was performed using standard GROMACS tools to calculate the root mean square deviation (RMSD).

### 4.21 Cell Thermal Shift Assay

The Cellular Thermal Shift Assay (CETSA) was performed to evaluate the direct binding between geldanamycin and caspase‐3 in a cellular context, as previously described [33]. Briefly, 293T cells transfected with wild‐type caspase‐3 or mutant caspase‐3 (R207A) plasmids were treated with 100 mM geldanamycin for 4 h. The cells were then harvested and resuspended in PBS containing a protease inhibitor cocktail. Following resuspension, the cells were subjected to heat shock in a thermal cycler for 3 min at temperatures ranging from 37°C to 72°C to induce protein denaturation, and immediately cooled on ice. Subsequently, all samples underwent three freeze‐thaw cycles using dry ice and a thermal cycler to ensure cell lysis. The lysates were centrifuged at 15 000 × g for 30 min at 4°C to remove cell debris and aggregated proteins. The resulting supernatant was boiled and analyzed by Western blotting. Protein bands were quantified using ImageJ software.

### 4.22 Drug Affinity Reactive Target Stability

The DARTS assay was conducted based on established protocols [33]. Briefly, cells were lysed using RIPA buffer (Beyotime, P0013) supplemented with protease and phosphatase inhibitor cocktails. The lysates were then supplemented with 10X TNC buffer (50 mM Tris‐HCl pH 8.0, 50 mM NaCl, 10 mM CaCl_2_), and total protein concentration was quantified using a BCA assay. Subsequently, cell lysates were incubated with increasing concentrations of geldanamycin or an equivalent volume of DMSO (vehicle control) for 1 h at room temperature. Following incubation, samples were digested with pronase (Roche, 10165921001) at a 1:5000 dilution for geldanamycin‐treated samples for 30 min at room temperature. The proteolytic reaction was terminated by boiling in SDS loading buffer. Finally, Western blotting was performed to evaluate whether geldanamycin directly interacts with fumarate. β‐Actin was used as a negative control.

### 4.23 Generation and Activation of Mouse BMDMs

The generation and activation of mouse BMDMs were performed following an established protocol [34]. To induce M2Φ polarization, BMDMs were treated with either 20 ng·mL^−^^1^ IL‐4 or 20 ng·mL^−^^1^ IL‐13. All cytokines were obtained from PeproTech (Rocky Hill, NJ, USA).

### 4.24 Transcriptomic Sequencing and Bioinformatic Analysis

Transcriptomic sequencing and subsequent bioinformatic analysis were conducted by Wuhan MetWare Biotechnology Co., Ltd. on total RNA extracted from murine LLC and RAW264.7 cell lines treated with geldanamycin (or an equivalent volume of DMSO as vehicle control) for 24 h. RNA was isolated using trizol reagent, with its quality and integrity rigorously assessed to ensure an RNA Integrity Number (RIN) greater than 7.0 prior to library construction. Sequencing libraries were prepared from enriched mRNA using oligo(dT) beads following the standard Illumina protocol for cDNA synthesis, end repair, A‐tailing, and adapter ligation. The resulting libraries were sequenced on an Illumina NovaSeq 6000 platform to generate 150 bp paired‐end reads. Raw sequencing data were processed to remove adapters and low‐quality sequences, yielding high‐quality clean data that were aligned to the murine reference genome (GRCm39) for quantification of gene expression levels, which were normalized and reported as FPKM. Differential gene expression analysis between geldanamycin‐treated and DMSO control groups was performed using DESeq2, with genes exhibiting an absolute log2 fold change greater than 1 and an adjusted p‐value less than 0.05 considered significantly differentially expressed.

### 4.25 Isolation of MPs

Lewis cells were plated into 10 cm cell culture dishes and irradiated with a single dose of 20 Gy by 6‐MV x‐rays. Then the medium was replaced with complete medium. After 72 h, the medium was collected and centrifuged at 1000 g for 10 min and then 14 000 g for 2 min. Next, the supernatant was centrifuged at 14 000 g for 1 h at 4°C to isolate MPs. Electroporation was used to load the geldanamycin onto MPs. The precipitate was washed with sterile 1× PBS twice and resuspended in sterile 1× PBS for animal experiments or resuspended in completed medium for cell experiments.

### 4.26 Quantification of MPs

The method of quantifying MPs is consistent with that of protein quantification. MPs were quantified with the BCA Protein Assay Kit (Thermo Fisher Scientific) according to the manufacturer's protocol.

### 4.27 Liquid Chromatography (HPLC)

Ultrasound of GA@P&S MPs was performed in methanol solution following centrifugation (14 000 g, 10 min). Then, the supernatants were filtered (0.2 µm filters) for HPLC (LC‐2030C Plus, designed by Shimadzu Corporation in Japan). AC18 (250 × 4.6 mm, 5 µm particle size) HPLC packed column was used as the chromatographic column. The mobile phase was CH_3_OH 0.5% TFA/H_2_O 0.5% TFA (1:1, v/v), the flow rate was 1.0 mL min^−1^, and the detection wavelength was 305 nm.

### 4.28 Characterization of MPs Size and Transmission Electron Microscopy Determination

One milliliter of MPs at a concentration of 20 ng·mL^−^^1^ was used to measure particle size and polydispersity index using a Malvern Zetasizer Nano ZSP laser particle size analyzer. For further characterization of size and morphology, the MPs were washed with ddH_2_O, deposited onto a copper mesh, and subsequently observed by TEM (HT7700‐SS or FEI Tecnai G20 TWIN).

### 4.29 Detection of Drug Release of MPs

To simulate a biologically relevant environment, MPs were incubated in mouse serum at 37 °C for varying durations. After incubation, the MPs were collected, washed, and subsequently analyzed for the concentrations of geldanamycin using HPLC and a microplate reader, respectively.

### 4.30 In Vitro Cellular Uptake Assay

To evaluate the cellular uptake of MPs, different cell lines were planted into 6‐well plates and incubated with DiD pre‐dyed MPs for 6 h. The cells were collected, washed by PBS and analyzed through flow cytometry (Beckman CytoFLEX S, USA).

### 4.31 Mice

C57BL/6 J mice (aged 5–6 weeks, weighted 18–20 g) were purchased from SHUBEILI Biotech. All mice were kept in micro‐isolator cages, and the experimental protocols were approved by the Hubei Provincial Animal Care and Use Committee and were in compliance with the experimental guidelines of the Animal Experimentation Ethics Committee of Huazhong Agricultural University (ID Number:202509110037).

### 4.32 Animal Experiments

A total of 2 × 10^6^ LLC cells were inoculated subcutaneously at the base of the thigh of C57BL/6 J mice. When the tumor volume was approximately 100 mm, 100 µg MPs were injected into the tumor of each mouse and administered once every other day for a total of four times. When the subcutaneous tumor volume is more than 1000 mm^3^, the mouse is recorded as dead at this point. The LLC cells 2 × 10^6^ cells suspended in 125 µL of PBS were injected via the tail vein to establish a mouse lung metastasis model. 12 days after inoculation with LLC cells, the mice were randomly grouped and received the respective treatments. The treatment methods included intravenous injection of 125 µL of liquid (once every 2 days, for a total of four times) or intraperitoneal injection of GA (equivalent to the dose in the GA@P&S MPs group, once every 2 days, for a total of four times). To establish the malignant pleural effusion (MPE) model, mice were anesthetized with 1% pentobarbital sodium prior to all procedures. LLC cells (500 000 cells suspended in 50 µL of PBS) were injected into the right pleural cavity through the 10th or 11th intercostal space at the midaxillary line. 4 days after LLC cell inoculation, the mice were randomly grouped and received corresponding treatments. The treatment methods included intrapleural injection of 50 µL of liquid (PBS or MPs suspension) under isoflurane anesthesia, administered once every 2 days for a total of four injections; followed by daily observation and recording of mouse survival.

### 4.33 Flow Cytometry

For cell‐surface analysis, cells were stained with the anti‐mouse Zombie NIR Fixable Viability Kit (BioLegend), CD45 (103114), CD11b (101263), F4/80 (123120), CD86 (105012), CD206(141706), CD11c(117311), CD64 (161004), CD103 (121423), MHCII (107664), Ly6G (108437), Ly6c (128031), CD3 (100212), CD4 (100408), and CD8a (100752) at the recommended concentrations at 4°C for 30 min. For T‐cells, GrazB (515406), IFN‐γ (505808), and FOXP3 (126408) cytokine staining, cells were fixed and permeabilized after stimulation with Phorbol 12‐myristate 13‐acetate (PMA) (ab120297, Abcam, 100 ng mL^−1^), Monensin sodium salt (ab120499, Abcam, 1 ug mL^−1^), and Ionomycin calcium salt (5608212, PeproTech, 100 ng mL^−1^) for 6 h. For CD206 (141706) staining, cells were also fixed and permeabilized. All flow cytometry antibodies were purchased from Biolegend (San Diego, CA, USA).

### 4.34 In Vivo Detection of MPs Phagocytosis

To examine the cellular uptake of MPs within the tumor microenvironment in vivo, we administered an intratumoral injection of 100 µL of DID labeled MPs into mice with LLC lung metastases. After 24 h, the mice were euthanized, and the tumors were dissociated into single‐cell suspensions for flow cytometry analysis. The cells were stained with the following antibodies: CD45 (157614), CD11b (101263), CD11c (117329). CD64 (161004), CD103 (121408), and CD326 (118206). Additionally, portions of the tumor tissue were fixed, dehydrated, and cryosectioned for preparation of frozen sections. These sections were subsequently immunostained with relevant antibodies and imaged using confocal laser scanning microscopy. To visualize the distribution of MPs, DiD‐stained MPs were observed under a fluorescence imaging system with an excitation filter of 540/20 nm and an emission filter of 620/20 nm, using an exposure time of 15 s.

### 4.35 Histology, Antibody Staining, and Imaging

To observe different immune cells within tumor tissues, LLC tumors and LLC lung metastases isolated from mice were frozen in Optimal Cutting Temperature (OCT) compound and sectioned. The sections were fixed with paraformaldehyde, then blocked with 1% BSA for 30 min at room temperature, and incubated overnight at 4°C with antibodies against F4/80, CD206, CD11c, MHCII, and IFN‐γ. After two washes with PBS, 4′,6‐diamidino‐2‐phenylindole (DAPI) was added to the slides to stain cell nuclei. For toxicity assessment, isolated organ tissues were fixed overnight in 4% paraformaldehyde, dehydrated, and embedded in paraffin. The tissue sections were then stained with H&E.

### 4.36 Statistical Analysis

The statistical differences between the two groups were analyzed using the statistics of the student’ t‐test, and the comparison between the three groups or more was carried out in two ways of ANOVA. Kaplan‐Meier survival curve of Malignant pleural effusion mouse was analyzed by Kaplan‐Meier statistical method.

## Author Contributions

These authors contributed equally: Tianli Hao, Zihan Deng, and Yufei Liu. T. Hao, Z. Deng, and Y. Liu performed the experiments and analyzed the data. D. Deng, M. Yang, and Y. Geng assisted with experimental design and data interpretation. L. Liu provided technical support and resources. Y. Liu, L. Lu, and H. Jin conceived the study, supervised the project, and acquired funding. T. Hao and Z. Deng wrote the original draft. Y. Liu, L. Lu, and H. Jin reviewed and edited the manuscript. All authors discussed the results and approved the final version of the manuscript.

## Funding

This study was supported by the National Natural Science Foundation of China (Grant No. 82272851, 82473363, 82402465), Fundamental Research Funds for the Central Universities (Program No. 2662024JC005), and the Hubei Provincial University Excellent Young and Middle‐aged Scientific and Technological Innovation Team Plan Project (Grant No. T2024033).

## Conflicts of Interest

The authors declare no conflict of interest.

## Supporting information




**Supporting file 1**: advs74886‐sup‐0001‐SuppMat.docx

**Supporting file 2**: advs74886‐sup‐0002‐Movie S1.avi

## Data Availability

The data that support the findings of this study are available from the corresponding author upon reasonable request.
