# Supplementary material for: Pyroptosis‐Inducing Engineered Microparticles for Cancer Immunotherapy
Source: Adv Sci (Weinh). 2026 Mar 18;13(31):e74886. doi: 10.1002/advs.74886 (PMC13587975; doi:10.1002/advs.74886)
Supplement: Supplementary file 1 [file ADVS-13-e74886-s002.docx]

**Supporting information**

**Pyroptosis-inducing Engineered Microparticles for Cancer Immunotherapy**

Tianli Hao^a,#^, Zihan Deng^a,#^, Yufei Liu^a,#^, Li Liu^a^, Deqiang Deng^b^, Muyang Yang^b^, Yuanyuan Geng^a^, Yao Sun^a^, Beilei Yue^a^ , Jonathan F. Lovell^c^, Yushuai Liu^d*^, Lisen Lu^b*^, Honglin Jin^a, b*^

^a^College of Biomedicine and Health and College of Life Science and Technology, Huazhong Agricultural University, Wuhan, 430070, China

^b^NHC Key Laboratory of Tropical Disease Control, School of Life Sciences and Medical Technology, Hainan Medical University, Haikou, Hainan, 571199, China

^c^Department of Biomedical Engineering, University at Buffalo, State University of New York, Buffalo, NY 14260, USA

^d^Department of Ophthalmology, Union Hospital, Tongji Medical College, Huazhong University of Science and Technology, Wuhan, 430022, China

^#^ Co-first authors: Tianli Hao, Zihan Deng and Yufei Liu, contributed equally to the article

^*^ Correspondence: jin@hust.edu.cn (Honglin Jin), lulisen@mail.hzau.edu.cn (Lisen Lu), liuyushuai1991@163.com (Yushuai Liu)

Figure S1. Analysis of the correlation between pyroptosis-related molecules and patient prognosis, as well as immune regulatory characteristics by using online lung cancer databases. (A) Identify the expression of GSDMA, GSDMB, GSDMC, GSDMED and GSDME gene between lung cancer and adjacent normal tissue in the TCGA database. (B) Kaplan-Meier curves of GSDMA, GSDMB, GSDMC, GSDMED and GSDME genes in lung cancer patients. (C) Identify the expression of GSDME gene between tumors and adjacent normal tissue in the TCGA database. (D-H) Kaplan-Meier curves of genes that regulate the activity of GSDME in lung cancer patients. (I) UMAP plot displaying the major cell types distribution in lung cancer patients. (J) Violin plot showed marker genes' expression levels in each cell type. (K) Identify the expression distribution of GSDME in each cell within different cell subtypes. (L) Identify ligand-receptor interactions related to cancer cell communication in the two lung cancer tumor cell clusters of GSDEM^high^Cas4^low^ and GSDEM^high^Cas4^high^. (M) Identify ligand-receptor interactions related from cancer cell communication in the two lung cancer tumor cell clusters of GSDEM^high^Cas4^low^ and GSDEM^high^Cas4^high^.

Figure S2. Cell survival rates following treatment with hit compounds from a pyroptosis drug screen. A representative bar chart showing the cell survival rates, as determined by CCK-8 assay, after treatment with the 19 final candidate compounds. These compounds were identified through three sequential rounds of screening (Primary: 139 hits (A); Secondary: 50 hits (B); Tertiary: 19 hits (C)).

Figure S3. Screening for small-molecule drugs that induce pyroptosis and identification of related functional targets. (A)The 19 small molecule candidates identified in Fig. S1 were further evaluated using a Hoechst/PI double-staining assay. Eight of these compounds were identified as potential inducers of pyroptosis. (B)The expression of GSDME protein in mouse cell lines by Western Blot. (C) Statistical analysis of the expression of GSDME in seven mouse cell lines. n=3 biologically independent samples. (D) Western Blot was used to determine the efficiency of GSDME knockdown in LLC cells using shRNA-GSDME. (E) The LDH detection kit was used to analyze the characteristics of LDH release in normal LLC cells, LLC cells with downregulated GSDME expression (shG-LLC), and LLC cells with overexpressed GSDME (G-LLC) induced by GA. n=3 biologically independent samples. (F) Flow cytometry was used to detect and statistically analyze the apoptosis efficiency of GA-induced LLC cells, shG-LLC cells, and G-LLC cells. n=3 biologically independent samples. (G) Hoechst/PI double-staining assay was used to detect and statistically analyze the apoptosis efficiency of GA-induced LLC cells, shG-LLC cells, and G-LLC cells. (H) Western Blot identified that the activity of GA in cleaving Caspase-3 is independent of the regulation of HSP90 activity. (I) Molecular dynamics simulation of the GA–caspase-3 interaction. The stable binding conformation and key residues between GA and caspase-3 were analyzed via molecular dynamics simulations, supporting their direct molecular interaction. (J) Analyze the relative intensity of Caspase-3 in Figure 1K and identify the thermal stability of GA-mediated Caspase-3 and mutant Caspase-3 in LLC through linear fitting curves. Data are presented as mean ± SEM, and statistical significance was analyzed *via* one-way ANOVA. *P <0.05, **P <0.01, ***P <0.001, and NS: not significant.

Figure S4. The flow cytometry gating strategy for different immune cells in Figure 2E-L (A) and Figure 2P-U (B).

Figure S5. Representative flow cytometry pseudocolor plots of data shown Figure 2E-L (A) and Figure 2P-U (B).

Figure S6. Biochemical toxicity assessment of mice treated with different concentrations of GA *in vivo*. (A) The percentage of lymphocytes, monocytes, and neutrophils, as well as hematocrit and red blood cell distribution width (RDW) in whole blood were measured for evaluation. n=3 biologically independent samples. (B) The number of white blood cells (WBC), neutrophils, lymphocytes, monocytes, red blood cells (RBC), and platelets in whole blood were measured for evaluation. n=3 biologically independent samples. (C) The levels of Albumin (ALB), alanine transaminase (ALT), aspartate transaminase (AST), creatinine (CREA) and UREA in serum for the evaluation of hepatic and renal function. Treatment with a high dose of geldanamycin significantly increased the levels of ALT and AST, indicating potential hepatotoxicity. n=3 biologically independent samples. (D) Histopathological examination of heart, liver, spleen, lung, and kidney in mice treated with different concentrations of GA. The Scale bar is 500 μm. Data are presented as mean ± SEM, and the statistical significance was analyzed *via* one-way ANOVA. **P* <0.05, ***P* <0.01, ****P* <0.001, and NS: not significant.

Figure S7. Characterization of the functional properties of GA@P&S-MPs and evaluation of their TIME targeting characteristics *in vivo.* (A) Western Blot identified that the engineered MPs were loaded with nPD-L1 or nSIRPα. The anti-PD-L1 nanobody was fused with RFP protein, and the anti-SIRPα nanobody was fused with GFP protein. The expression of RFP or GFP was detected to characterize the expression of nPD-L1 or nSIRPα. (B) Flow cytometry was used to analyze the nPD-L1 or nSIRPα carried by different engineered MPs. (C) The relative viability of LLC cells treated with indicated treatment was detected by CCK8 assay. n=3 biologically independent samples. The percentage of Annexin V^+^/PI^+^ in LLC cells (D) and the LDH level from the supernatant (E) treated with indicated treatment were measured. n=3 biologically independent samples. (F) Flow cytometry was used to evaluate the relative targeting ability of different engineered MPs to RAW264.7 cells. The indicated MPs were stained with DiD. n=3 biologically independent samples. (G) Flow cytometry was used to analyze the polarization of macrophages by evaluating the expression levels of CD86 in BMDM cells. n=3 biologically independent samples. (H) The flow cytometry gating strategy for different immune cells in Lung TIME, containing tumor cell, DCs and macrophage. (I-K) The phagocytosis of the MPs by DCs (I), macrophages (J), and tumor cells (K) were analyzed by flow cytometry. n=3 biologically independent samples. Data are presented as mean ± SEM, and the statistical significance was analyzed *via* one-way ANOVA. **P* <0.05, ***P* <0.01, ****P* <0.001, and NS: not significant.

Figure S8. The flow cytometry gating strategy for different immune cells in Figure 6D-K (A) and Figure 7G-N (B).

Figure S9. Representative flow cytometry pseudocolor plots of data shown Figure 6D-K.

Figure S10. Representative flow cytometry pseudocolor plots of data shown Figure 7G-N.

Figure S11. *In vivo* toxicity assessment indicated engineered MPs. (A)The percentage of lymphocytes, monocytes, and neutrophils, as well as hematocrit and red blood cell distribution width (RDW) in whole blood were measured for evaluation. n=3 biologically independent samples. (B)The number of white blood cells (WBC), neutrophils, lymphocytes, monocytes, red blood cells (RBC), and platelets in whole blood were measured for evaluation. n=3 biologically independent samples. (C)The levels of Albumin (ALB), alanine transaminase (ALT), aspartate transaminase (AST), creatinine (CREA) and UREA in serum for the evaluation of hepatic and renal function. n=3 biologically independent samples. (D) Histopathological examination of heart, liver, spleen, lung, and kidney. The scale bar is 100 μm. Data are presented as mean ± SEM, and the statistical significance was analyzed *via* one-way ANOVA. **P* <0.05, ***P* <0.01, ****P* <0.001, and NS: not significant.

Table 1：The amino acid sequences of the fusion proteins and the primer sequences involved in the study.

| Segment |  | Sequence |
| --- | --- | --- |
| GSDME | Forward | 5'- gatctggcccgggcgggatccATGTTTGCCAAAGCAACTCGA -3' |
|  | Reverse | 5'- gtcatccttgtaatcctcgagGTCTTGACCTGTAGCATGTCCTGA -3' |
| GSDMD | Forward | 5'- gatctggcccgggcgggatccATGCCATCGGCCTTTGAGA -3' |
|  | Reverse | 5'- gtcatccttgtaatcctcgagACAAGGTTTCTGGCCTAGACTTGA -3' |
| shGSDME |  | 5'- GACTTGCTGCAAACTCCATGT -3' |
| Caspase3 | Forward | 5'- GCGGATCGGGTTTAAACggatccATGGAGAACAACAAAACCTCAGTG-3' |
|  | Reverse | 5'- ATCGGGCCCTCTAGActcgagGTGATAAAAGTACAGTTCTTTCGTGAGC-3' |
| Caspase3 R207A | Forward | 5'-CACCTGGTTACTATTCCTGGGCAAATTCAAAGGACGGGTCG-3' |
|  | Reverse | 5'-CGACCCGTCCTTTGAATTTGCCCAGGAATAGTAACCAGGTG-3' |
| CD8α-PD-L1Nb-GPI |  | MRLTVGALLACAALGLCLAGGGGSGGGGSGGGGSMAQVQLVETGGGLVQPGGSLRLSCTASGFTFSMHAMTWYRQAPGKQRELVAVITSHGDRANYTDSVRGRFTISRDNTKNMVYLQMNSLKPEDTAVYYCNVPRYDSWGQGTQVTVSSGGGGSGGGGSGGGGSGGSSLQSTAGLLALSLSLLHLYC |
| CD8α-SIPRαNb-GPI |  | MRLTVGALLACAALGLCLAGGGGSGGGGSGGGGSQVQLQESGGGSVQAGGSLRLSCATSGLTYSAYCMGWFRQAPGKGRETVAQINLGDGDTYYADSVKGRFAISQDNAKNTVHLLMNGLKPEDTAIYYCAGSDHMCAKWHYWGQGTQVTVSSGGGGSGGGGSGGGGSGGSSLQSTAGLLALSLSLLHLYC |
